# Effect of Solvents on Electrogenerated Base-Driven Transfer Hydrogenation Reactions

**DOI:** 10.3390/molecules30040910

**Published:** 2025-02-15

**Authors:** Jing-Wei Zhu, Meng-Han Li, Feng Zhang, Ya-Li Wang, Jia-Xing Lu, Huan Wang

**Affiliations:** 1 Key Laboratory of Petroleum Molecular & Process Engineering, Shanghai Key Laboratory of Green Chemistry and Chemical Processes, School of Chemistry and Molecular Engineering, East China Normal University, Shanghai 200062, China; 52214300053@stu.ecnu.edu.cn (J.-W.Z.); 51254300102@stu.ecnu.edu.cn (M.-H.L.); 51264300109@stu.ecnu.edu.cn (F.Z.); wyl198701@163.com (Y.-L.W.); jxlu@chem.ecnu.edu.cn (J.-X.L.); 2 College of Chemistry and Chemical Engineering, Ningxia Normal University, Guyuan 756099, China; 3 Institute of Eco-Chongming, 20 Cuiniao Road, Chenjia Town, Chongming District, Shanghai 202162, China

**Keywords:** transfer hydrogenation, electrogenerated base, solvent effect, manganese catalysts, aromatic ketones

## Abstract

Transfer hydrogenation is a crucial technology for synthesizing fine chemicals and pharmaceuticals, offering improved safety and convenience over traditional hydrogen methods, although it typically requires external bases. While isopropanol is commonly used as a hydrogen source, methanol is superior but faces challenges due to its high dehydrogenation energy barrier, limiting its use under mild conditions. This study focuses on investigating the differences in the electrogenerated base-driven transfer hydrogenation of aromatic ketones in isopropanol and methanol solvents, using Mn(CO)₅Br and cyclohexanediamine derivatives as the catalyst. The research demonstrates that high enantiomeric excess (ee) values were obtained in isopropanol in the presence of chiral Mn-based catalysts, while only racemic products were observed in methanol. The results indicate a strong dependence of the catalytic pathway on the choice solvent: in isopropanol, the catalyst operates via a metal–ligand cooperative transfer hydrogenation, resulting in high ee values, whereas in methanol, transfer hydrogenation occurs through metal hydride transfer with no stereoselectivity.

## 1. Introduction

Compared to the traditional hydrogenation method using molecular hydrogen (H_2_), transfer hydrogenation has emerged as a key technology for the synthesis of fine chemicals and pharmaceuticals, offering higher safety, more convenient operation, and lower equipment requirements [1,2,3]. This method is commonly used for the reduction of substances such as ketones [4,5], imines [6], and nitro compounds [7]. Among them, ketones are particularly noteworthy because their reduction products, alcohols, are among the most widely used industrial raw materials and serve as important precursors for other drug molecules [8,9].

Since Henbest and Mitchell first reported the use of isopropanol as the hydrogen source for transfer hydrogenation in 1964 [10], significant progress has been made over the decades. Transition metal catalysts have become popular due to their high efficiency [11,12], stability [13,14,15], and flexible ligand design [16,17]. Among them, Ru-based catalysts are the most commonly used in reaction systems with isopropanol as both solvent and hydrogen source [18,19,20,21]. Since the first Mn-based catalyst was confirmed to be effective for the transfer hydrogenation of aromatic ketones in 2017 [22], Mn-based catalysts have garnered attention for their cost-effectiveness and efficiency comparable to Ru-based catalysts [23,24,25,26]. Sortais et al. [27] studied in detail the effects of different ligands on the transfer hydrogenation of aromatic ketones using isopropanol as the hydrogen source. The combination of chiral diamine ligands with Mn(CO)_5_Br produced chiral products with good enantiomeric excess (ee) values, whereas the use of non-chiral diamine ligands produced racemic products. Pidko et al. [28] then used DFT calculations to investigate asymmetric transfer hydrogenation mechanisms with simple Mn–diamine catalysts.

However, conventional transfer hydrogenation reactions using transition metal catalysts often require the addition of external bases to drive the catalytic reaction [29,30,31]. The need for external bases can complicate industrial production and potentially inter-fere with product separation. The use of electrochemical methods [32] to generate bases during the reaction, i.e., electrogenerated bases (EGBs) [33], could be more advantageous. As a green technology, EGBs can be generated in situ during the reaction, and their amount can be controlled by adjusting the amount of electricity [34]. Recently, our group demonstrated that electrogenerated bases could be an effective alternative to external bases in the transfer hydrogenation of aromatic ketones in an ethanol solution [35]. Methanol is a low-cost and safe solvent, considered an ideal hydrogen source; however, relevant cases are limited [36,37,38]. Moreover, in traditional organic reactions, different solvents can significantly influence the catalytic mechanisms of catalysts, indicating that solvents are a key factor in the reaction [39,40]. Due to the scarcity of cases involving methanol as a hydrogen source in the transfer hydrogenation of aromatic ketones, research on the effects of methanol compared to the classical solvent isopropanol on this process is notably rare.

In this work, we have used electrogenerated bases to drive catalysts consisting of cyclohexanediamine derivatives and Mn(CO)_5_Br, and investigated the effect of isopropanol or methanol as both solvent and hydrogen source on the transfer hydrogenation of aromatic ketones.

## 2. Results and Discussion

### 2.1. Reaction Optimization and Cyclic Voltammetry

Previous work [35] has shown that ethanol can not only act as a solvent in electrolysis, but also produce a Lewis base, which enables metal–organic complexes to catalyze the asymmetric transfer hydrogenation of aromatic ketones. To explore the effect of solvents, here, isopropanol (i-PrOH) was chosen as both the solvent and hydrogen source, Et_4_NBF_4_ as the electrolyte, Mn(CO)_5_Br along with (1R,2R)-N1,N2-dimethylcyclohexane-1,2-diamine (**L1**) as the catalyst, platinum foil as the cathode, and a magnesium rod as the anode in the preliminary reaction to study the transfer hydrogenation of acetophenone. Under this condition, the selectivity (isolate yield/conversion) of 1-phenylethanol was 99%, with an ee value of 63% (Table 1 entry 1). As the temperature decreased gradually from 60 °C to 20 °C, the selectivity remained unchanged and the ee value increased slightly; however, the conversion decreased significantly (Table 1, entries 1–3). The decrease in conversion indicates that the reaction rate slows down at lower temperatures. Meanwhile, the slight increase in ee value suggests that low temperature is conducive to the formation of chiral products, which may be related to the change in the stability of the reaction intermediates or the alteration in the reaction kinetics [28]. When neither the ligand nor Mn(CO)_5_Br, or only one of them, was present in the electrolyte, almost no product was detected (Table 1, entries 4–6). Similarly, when no current was passed through the reaction system, no products were detected (Table 1, entry 7). These results indicate that the reaction requires the simultaneous presence of Mn(CO)_5_Br, a ligand, and electricity. Replacement of the chiral ligand with an achiral ligand, N1,N2-dimethylcyclohexane-1,2-diamine (**L2**), gave only racemic products, although the conversion and selectivity remained nearly unchanged (Table 1, entry 8). This suggests that the chirality of the product is closely related to the chirality of the ligand.

**Figure 1:**
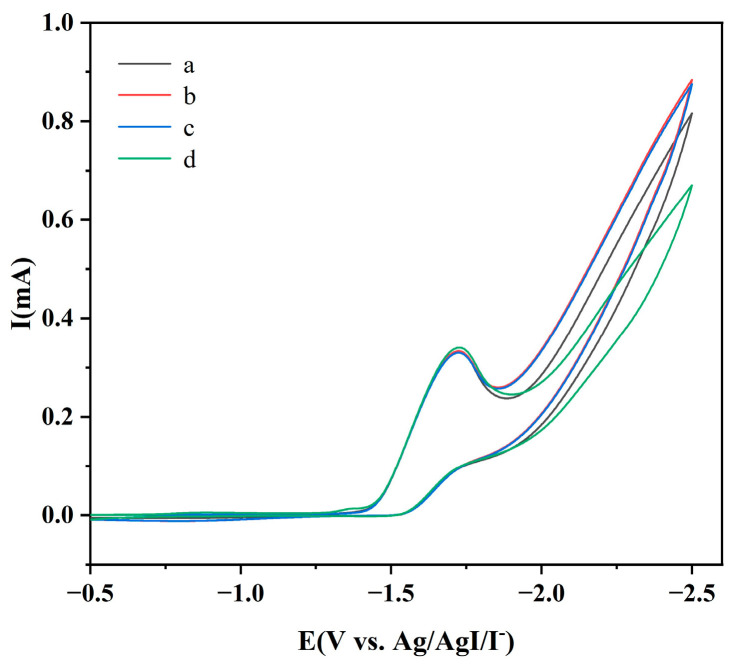
CV curves recorded in DMF containing 0.05 M Et_4_NBF_4_: (a) 10 mM acetophenone, (b) as a + 1.125 mol% **L1**, (c) as a + 0.75 mol% Mn(CO)_5_Br, (d) as b + 0.75 mol% Mn(CO)_5_Br.

The above electrolysis results show that i-PrOH can be used as a solvent for the electrochemical hydrogenation of acetophenone. However, is this reaction process similar to previous reports [21,35], which are also driven by an electrogenerated base? Does the metal complex catalyst undergo an electron transfer process? To answer these questions, cyclic voltammetry (CV) was employed to study the electrochemical behavior of the reaction system. To avoid the interference of the polarization effect of the solvent, i-PrOH, on the CV curves, DMF was first chosen as the solvent for the test. As can be seen from Figure S1, no redox peaks could be detected in the scanned potential ranges regardless of whether the solution contained only **L1,** or Mn(CO)_5_Br, or both coexisted, indicating that it was difficult for these compounds to gain or lose electrons within their corresponding potential range. Additionally, when equimolar amounts of acetophenone were added to the systems containing **L1**, Mn(CO)_5_Br, and a mixture of **L1** and Mn(CO)_5_Br, respectively, the reduction peaks were almost identical to that in the solution containing only acetophenone (Figure 1). This indicates that the presence or absence of a metal complex catalyst does not affect the electron transfer reaction between acetophenone and the cathode. Moreover, no significant redox peaks were detected with the addition of the substrate to the i-PrOH system, which may be due to the reduction peak of acetophenone being obscured by the polarization of the solvent (Figure S2). This indicates that i-PrOH is more easily reduced at the cathode than the substrate.

Then, comparative electrolysis experiments were conducted. A conversion of 57% and a selectivity of 97% were achieved by first electrolyzing the electrolyte and then adding the catalyst and substrate (Table 1, entry 9). When potassium tert-butoxide (equivalent to the electrogenerated base) was used directly in place of the electrogenerated base, the experimental results were almost identical to those obtained with the electrogenerated base (Table 1, entry 10). Based on the CV curves and electrolysis results, it was speculated that the isopropoxide anion generated from the reduction of i-PrOH creates an alkaline environment in i-PrOH, which drives the catalyst to perform the catalytic reaction.

When the solvent was changed to MeOH, the conversion was 65%, while the selectivity was 60%, but unexpectedly, the ee value was 0% (Table 2, entry 1). Different solvents have a certain impact on the selectivity and ee value of the products, but it is very rare for a change in solvent to result in the obtaining of racemic products instead of chiral products, either in conventional or electrogenerated base-driven organometallic catalytic systems. Even when changing the temperature and catalyst equivalents, only racemic products could be obtained, with a conversion of 68% and a selectivity of 79% after optimization (Table 2, entries 2–4). The electrochemical behavior of the substrate and solvent in MeOH is similar to that in i-PrOH (Figure S3). This suggests that the solvent, MeOH, is more easily reduced than the substrate. Similarly to the reaction in i-PrOH, no product was detected when no current passed through the reaction system (Table 2, entry 5). The difference, however, is that when potassium tert-butoxide (equivalent to the electrochemical base) was used instead of electrolysis, no product was obtained in the MeOH solution, and there was little conversion of the substrate (Table 2, entry 6). By reviewing the literature, it was found that it is very difficult to perform transfer hydrogenation with MeOH as both the solvent and hydrogen source, which may be due to the high C-H bond breaking energy barrier of MeOH [41,42]. Therefore, conducting transfer hydrogenation reactions in MeOH is quite challenging and often requires stringent reaction conditions. Compared to traditional organometallic catalytic transfer hydrogenation, electrogenerated base-driven transfer hydrogenation in MeOH requires much milder reaction conditions (Table S1) [9,41,43].

In both i-PrOH and MeOH solvents, the transfer hydrogenation of acetophenone can be achieved electrochemically. However, the differences in the chirality of the products in the two solutions indicate that their catalytic processes may differ.

### 2.2. Substrate and Ligand Scopes

**Scheme 1:**
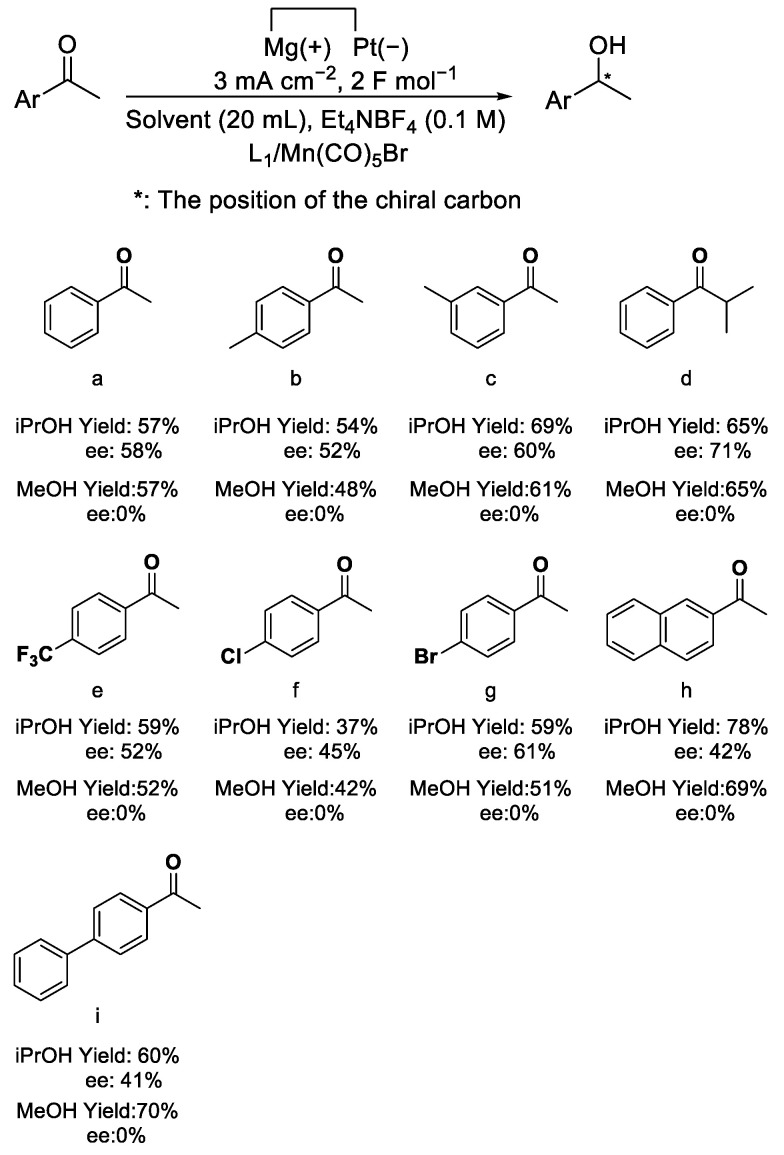
Substrate scope for transfer hydrogenation of ketones in i-PrOH and MeOH.

To explore whether the observed experimental phenomena were also influenced by factors other than the solvent, we examined the effects of substrates and ligands on the reaction. As shown in Scheme 1, except for phenylacetone, other common substrates, such as methyl phenylacetone, 2-methyl-1-phenylpropan-1-one, 4-halophenylacetone, 2-naphthylacetone, and biphenylacetone, all yielded products with high ee values in i-PrOH, while racemic products were produced in MeOH. This indicates that the substituents on the substrates have a minimal impact on the reaction outcomes.

**Scheme 2:**
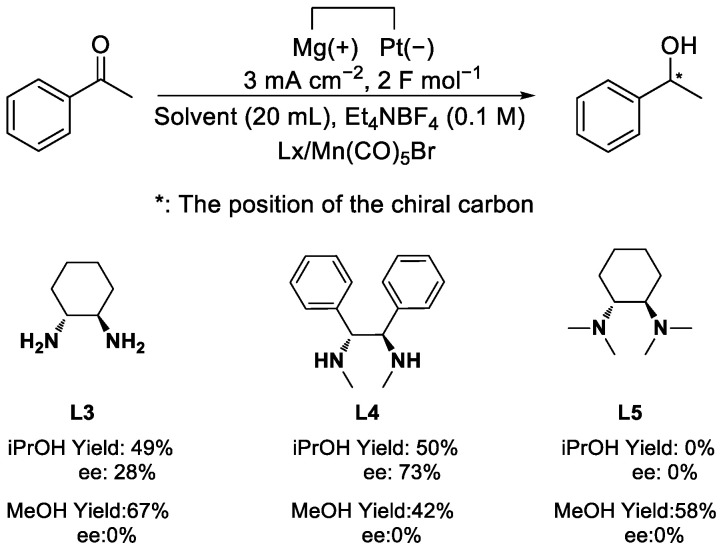
Ligand scope for transfer hydrogenation of acetophenone in i-PrOH and MeOH.

Then, we selected several ligands for further investigation. The data (Scheme 2) showed that, with the exception of **L5**, several other ligands gave similar results as before, and the chirality of the products was related to the alcohol solvent. Surprisingly, when **L5** was used in i-PrOH, no product was detected, while the conversion in MeOH reached 78% with a selectivity of 58% (Scheme 2). Apparently, **L5** can act as a good catalyst in MeOH but fails to promote transfer hydrogenation in i-PrOH. Structurally, **L5** is distinguished from other ligands, such as **L1**, by the absence or presence of hydrogen on the nitrogen atom of the ligand. The failure of **L5** in i-PrOH suggests that the involvement of the N-H bond in the ligand is essential for effective catalytic transfer hydrogenation in i-PrOH. Conversely, the catalytic process in MeOH does not appear to rely on the involvement of the N-H in the ligand. This suggests that the catalytic process in methanol may follow different pathways compared with the ligand–metal synergistic transfer hydrogenation in isopropanol.

### 2.3. UV-Vis Absorption Spectra

**Figure 2:**
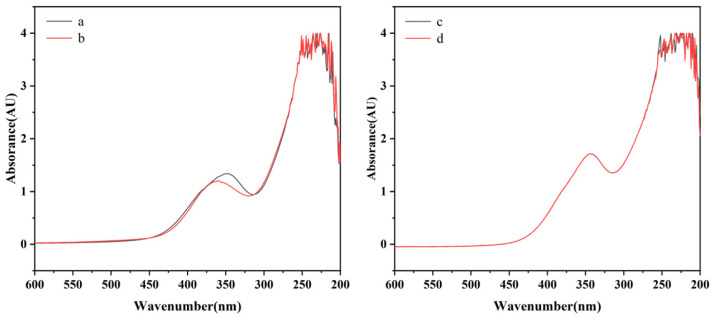
UV-Vis absorption spectra of Mn(CO)_5_Br/**L1**(1/1.5) in i-PrOH (**left**) and in MeOH (**right**) containing 0.05 M Et_4_NBF_4_, before (a, c) and after (b, d) addition of 10 μL of EGB solution.

In order to gain deeper insights into the differences in catalyst driving by electrogenerated bases in MeOH and i-PrOH, attempts were made to record the changes in catalysts in electrogenerated base systems using a UV–visible spectrophotometer. As shown in Figure 2 (left) the addition of electrolyzed i-PrOH to the i-PrOH solution containing Mn(CO)_5_Br and **L1** resulted in a significant red shift in the UV-Vis absorption spectrum of the catalyst, along with a decrease in peak intensity. This may be attributed to the addition of electrogenerated isopropoxide ions leading to the departure of HBr from the complex and the formation of metal–N double bonds, which increases the conjugation of the complex [14,35]. Additionally, when potassium tert-butoxide was used instead of the electrogenerated base, the changes observed in the UV-Vis spectra were consistent with those observed when the electrogenerated base was used (Figure S4). This implies that both bases might drive the catalytic reaction in similar ways in i-PrOH solution. In MeOH solution, on the other hand, the UV-Vis spectra of the catalyst showed no significant changes before and after the addition of electrolyzed MeOH (Figure 2 Right), which contrasts sharply with the changes in UV-Vis spectra in i-PrOH solution. The UV-Vis spectroscopy data indicate that the catalytic process in MeOH is likely different from that in i-PrOH.

### 2.4. Mechanism Studies

**Scheme 3:**
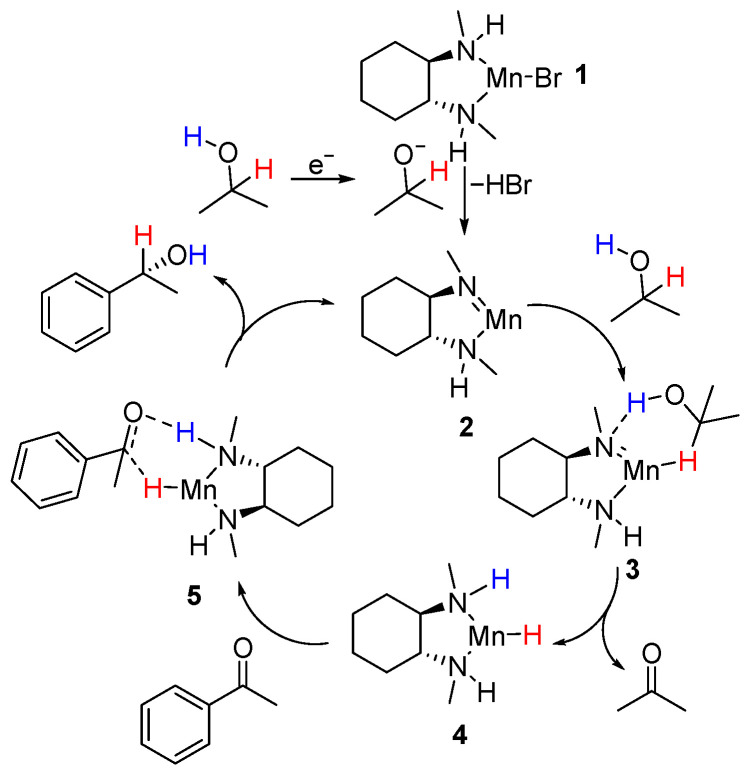
Possible reaction mechanism in i-PrOH.

Based on the above results, it can be postulated that the catalysts undergo different catalytic processes in the i-PrOH and MeOH solutions. In i-PrOH (Scheme 3), in the presence of electrogenerated isopropoxy anions, the catalyst **1** removes HBr to form complex **2** with a Mn=N double bond, which then reacts with i-PrOH to form intermediate **3**. Subsequently, accompanied by the departure of acetone, intermediate **4** is formed, which binds to the aromatic ketone in concert with the ligand and undergoes hydrogen transfer to produce the corresponding alcohol with a good ee value. This process is similar to that of organic base-driven transfer hydrogenation [28].

**Scheme 4:**
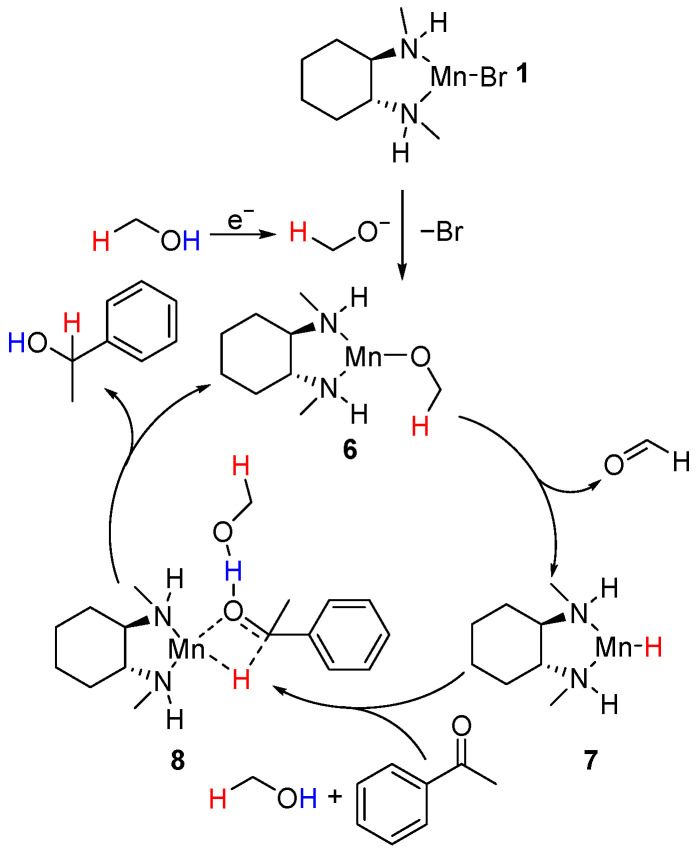
Possible reaction mechanism in MeOH.

Analysis of the UV-Vis results shows that the reaction in MeOH solution does not involve intermediate **2** containing the Mn=N bond. In electrochemical reactions, the electron transfer occurs at the electrode/electrolyte interface, so that the concentration of electrogenerated intermediates in this region tends to be significantly higher than that in the bulk solution. Therefore, in MeOH, it is possible that the high concentration of electrogenerated methoxy anions within the microenvironment of the electrode surface effectively promotes the catalyst to release the halogen anions, which in turn binds to the methoxy anion and undergoes the formation of intermediate **6** to form metal hydride **4** (Scheme 4). An isotopic experiment was performed using CH_3_-OD as the solvent and the hydrogen source. The NMR results show that the hydrogen on the α-C connected to the hydroxyl group in 1-phenylethanol is H rather than D (Figure S5). This suggests that the hydrogen on α-C likely originates from the methyl group of methanol. Moreover, relative to isopropanol, due to the higher reactivity of the hydroxyl hydrogen in methanol, it is more likely to bind to aromatic ketones during catalysis [44]. The methanol molecule occupies a certain space, which makes it more difficult for the hydrogen on the amine group to interact with the aromatic ketone, thus facilitating the formation of intermediate **7**. Based on the above electrolytic and UV–visible spectroscopic results, a notable feature of this catalytic process is the differential influence of the solvent on the chirality of the products.

**Figure 3:**
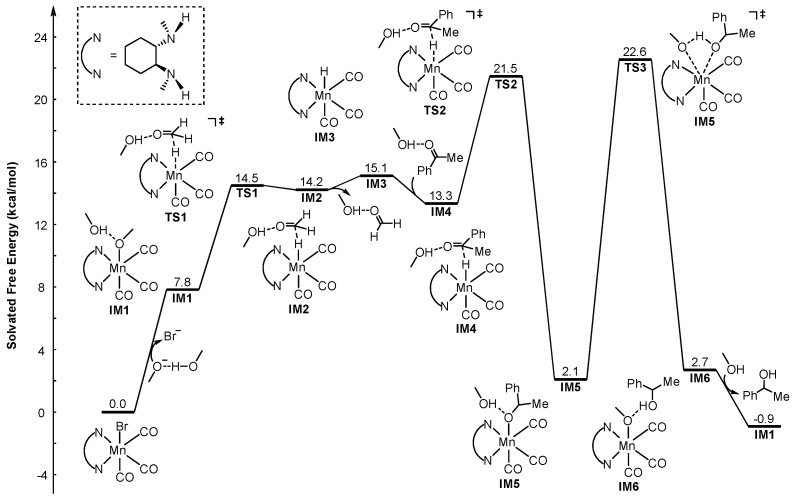
Calculated Gibbs free energies (kcal/mol) for the formation of 1-phenylethanol from acetophenone using methanol. (‡: transition state).

Based on the aforementioned information, experimental results, and characterization data, we proposed and analyzed a reasonable reaction mechanism in MeOH using Density Functional Theory (DFT) calculations (Figure 3) [45,46,47,48,49,50,51,52,53,54]. First, complex **1** reacts with the electrophilic Lewis base, where the halogen leaves, then the O in the Lewis base coordinates with the metal center, forming **IM1**. This step requires overcoming an energy barrier of 7.8 Kcal/mol. Then, β-hydrogen is eliminated by forming **TS1** (14.5 Kcal/mol), followed by the removal of formaldehyde after **IM2** to produce manganese hydride **IM3**, and one molecule of formaldehyde leaves. The substrate’s C=O double bond inserts into hydrogenated manganese forming **IM4**, then crosses an energy barrier of 8.2 kcal/mol to form **TS2**, followed by the formation of **IM5**. Subsequently, crossing a higher energy barrier of 20.5 kcal/mol leads to **TS3**, resulting in **IM6**, where finally the hydroxyl hydrogen from the methanol transfers onto the substrate to yield aromatic alcohol products. Based on the experimental results and DFT calculation data, the formation of racemic products in MeOH may be the result of the combined action of the electrogenerated base and solvation. The high concentration of methoxy anions generated at the cathode lowers the energy barrier for hydrogen transfer, facilitating the formation of metal hydrides. Meanwhile, the solvation effect shields the ligand when the substrate binds to the metal hydride, occupying space and hindering the synergistic metal–ligand interaction. As a result, the reaction in MeOH proceeds exclusively via the metal hydride catalysis pathway, leading to racemic products.

## 3. Experimental Section

### 3.1. General Information

Isopropanol (Sinopharm Chemical Reagent Co., Ltd. Shanghai, China), methanol (Sinopharm Chemical Reagent Co., Ltd.), tetraethylammonium tetrafluoroborate (Beijing Innochem Technology Co., Beijing, China), bromopentacarbonyl manganese(I) (Adamas Reagent Co. Shanghai, China), (1R,2R)-N1,N2-dimethylcyclohexane-1,2-diamine (**L1**) (Leyan Reagent Co. Shanghai, China), N1,N2-dimethylcyclohexane-1,2-diamine (**L2**) (Beijing Innochem Technology Co.), (1R,2R)-cyclohexane-1,2-diamine (**L3**) (Leyan Reagent Co.), (1R,2R)-N1,N2-dimethyl-1,2-diphenylethane-1,2-diamine (**L4**) (Beijing Innochem Technology Co.), (1R,2R)-N1,N1,N2,N2-tetramethylcyclohexane-1,2-diamine (**L5**) (Beijing Innochem Technology Co.), acetophenone (Sinopharm Chemical Reagent Co., Ltd.), 1-(p-tolyl)ethenone, 1-(4-chlorophenyl)ethenone (Beijing Innochem Technology Co.), 1-(4-(trifluoromethyl)phenyl)ethanone (Beijing Innochem Technology Co.), 1-(m-tolyl)ethanone (Beijing Innochem Technology Co.), 1-(naphthalen-2-yl)ethenone (Beijing Innochem Technology Co.), 1-(4-bromophenyl)ethenone (Beijing Innochem Technology Co.), 2-methyl-1-phenylpropan-1-one (Beijing Innochem Technology Co.), and 1-([1,1′-biphenyl]-4-yl)ethenone (Beijing Innochem Technology Co.) were obtained from commercial sources and used without further purification. All reactions and manipulations were carried out under an atmosphere of pure nitrogen using standard Schlenk techniques in oven-dried glassware. The solvents were purified by standard methods under dry nitrogen before use.

### 3.2. General Procedure for the Electrolysis

A typical galvanostatic electrolysis was carried out in a mixture of 2,2,2-trifluoroacetophenon (0.1 M), ligand (4.5 mol% in i-PrOH or 9.0 mol% in MeOH), Mn(CO)_5_Br (3.0 mol% in i-PrOH or 6.0 mol% in MeOH), Et_4_NBF_4_ (0.05 M), and solvent (i-PrOH or MeOH 20 mL) in a one-chamber electrolytic cell with a sacrificial Mg anode and a Pt cathode with a direct-current regulated power supply (HY3002D, HYelec, Shanghai, China). The enantiomeric excess (ee) value (Equation (S1)) was measured with HPLC with an O-DH column.

### 3.3. Procedure for UV-Vis Absorption Spectra

The spectra of the solution containing Mn(CO)_5_Br (1.5 mol%) and **L1** (2.25 mol%) were monitored by UV-Vis spectroscopy (G1103A, Agilent Technologies (China) Ltd., Shanghai, China), before and after the addition of the electrolyte.

### 3.4. Cyclic Voltammetry

Cyclic voltammograms were analyzed on a CHI 600c electrochemical station (Shanghai Chenhua Instruments Company, Shanghai, China) comprising a three-electrode cell equipped with platinum (Pt, d = 2 mm) as the working electrode, a Pt gauze (2 cm × 2 cm) as the counter electrode, and an electrode of Ag/AgI/0.05 M Bu_4_NI in DMF as the reference electrode. All electrochemical measurements were performed at a scan rate of 0.1 V s^−1^ in a N_2_-saturated solution.

### 3.5. Characterization Data for Product

1-phenylethanol. Eluent: ethyl acetate/petroleum ether = 1:10 *v*/*v*. Light yellow oil (142 mg, 58%). ^1^H NMR (500 MHz, CDCl_3_, δ ppm): 7.43–7.27 (m, 5H), 4.87 (q, *J* = 6.5 Hz, 1H), 2.76 (s, 1H), 1.50 (d, *J* = 6.5 Hz, 3H). ^13^C NMR (500 MHz, CDCl_3_, δ ppm): 145.8, 128.5, 127.5, 125.4, 70.4, 25.1.

1-(p-tolyl)ethanol. Eluent: ethyl acetate/petroleum ether = 1:10 *v*/*v*. Light yellow oil (147 mg, 54%). ^1^H NMR (500 MHz, CDCl_3_, δ ppm): 7.29 (d, *J* = 10.0 Hz, 2H), 7.20 (d, *J* = 10.0 Hz, 2H), 4.87 (m, 1H), 2.38 (m, 4H), 1.51 (d, *J* = 5.0 Hz, 3H). ^13^C NMR (500 MHz, CDCl_3_, δ ppm): 143.1, 137.1, 129.2, 125.4, 70.2, 25.1, 21.2.

1-(m-tolyl)ethanol. Eluent: ethyl acetate/petroleum ether = 1:10 *v*/*v*. Light yellow oil (188 mg, 59%). ^1^H NMR (500 MHz, CDCl_3_, δ ppm): 7.30–7.10 (m, 4H), 4.88 (m, 1H), 2.40 (s, 3H), 2.07 (s, 1H), 1.51 (d, *J* = 5.0 Hz, 3H). ^13^C NMR (500 MHz, CDCl_3_, δ ppm): 145.8, 138.2, 128.3, 128.2, 126.1, 122.5, 70.4, 25.1, 21.5.

2-methyl-1-phenylpropan-1-ol. Eluent: ethyl acetate/petroleum ether = 1:10 *v*/*v*. Light yellow oil (195 mg, 65%). ^1^H NMR (500 MHz, CDCl_3_, δ ppm): 7.40–7.28 (m, 5H), 4.36 (d, *J* = 10 Hz, 1H), 2.15 (s, 1H), 2.03–1.90 (m, 1H), 1.03 (d, *J* = 5.0 Hz, 3H), 0.83 (d, *J* = 5.0 Hz, 3H). ^13^C NMR (500 MHz, CDCl_3_, δ ppm): 143.7, 128.2, 127.5, 126.7, 80.1, 35.3, 19.0, 18.3.

1-(4-(trifluoromethyl)phenyl)ethanol. Eluent: ethyl acetate/petroleum ether = 1:10 v/v. Light yellow oil (224 mg, 59%). ^1^H NMR (500 MHz, CDCl_3_, δ ppm): 7.60 (d, *J* = 8.1 Hz, 2H), 7.45 (d, *J* = 8.1 Hz, 2H), 4.91 (t, *J* = 9.7 Hz, 1H), 2.83 (s, 1H), 1.48 (d, *J* = 6.5 Hz, 3H). ^13^C NMR (500 MHz, CDCl_3_, δ ppm): 149.7, 129.7(q, *J* = 125 Hz, -CF_3_), 125.6, 125.4, 123.1, 69.7, 25.2.

1-(4-chlorophenyl)ethanol. Eluent: ethyl acetate/petroleum ether = 1:10 *v*/*v*. Light yellow oil (115 mg, 37%). ^1^H NMR (500 MHz, CDCl_3_, δ ppm): 7.31 (m, 4H), 4.83 (s, 1H), 2.61 (m, 1H), 1.45 (s, 3H). ^13^C NMR (500 MHz, CDCl_3_, δ ppm): 144.3, 133.2, 128.6, 126.8, 69.7, 25.2.

1-(4-bromophenyl)ethanol. Eluent: ethyl acetate/petroleum ether = 1:10 *v*/*v*. Light yellow solid (237 mg, 59%). ^1^H NMR (500 MHz, CDCl_3_, δ ppm): 7.43 (d, *J* = 10.0 Hz, 2H), 7.16 (d, *J* = 10.0 Hz, 2H), 4.74 (m, 1H), 3.35 (s, 1H), 1.40 (d, *J* = 5.0 Hz, 3H). ^13^C NMR (500 MHz, CDCl_3_, δ ppm): 144.8, 131.5, 127.2, 121.0, 69.6, 25.2.

1-(naphthalen-2-yl)ethanol. Eluent: ethyl acetate/petroleum ether = 1:10 *v*/*v*. Light yellow solid (268 mg, 78%). ^1^H NMR (500 MHz, CDCl_3_, δ ppm): 7.90–7.81 (m, 4H), 7.52 (m, 3H), 5.08 (m, 1H), 2.09 (s, 1H), 1.61 (d, *J* = 5.0 Hz, 3H). ^13^C NMR (500 MHz, CDCl_3_, δ ppm): 143.2, 133.4, 133.0, 128.3, 128.0, 127.7, 126.2, 125.8, 123.9, 70.5, 25.2.

1-([1,1′-biphenyl]-4-yl)ethanol. Eluent: ethyl acetate/petroleum ether = 1:10 *v*/*v*. Light yellow solid (238 mg, 60%). ^1^H NMR (500 MHz, CDCl_3_, δ ppm): 7.62 (d, *J* = 10.0 Hz, 4H), 7.51–7.44 (m, 4H), 7.38 (t, *J* = 5.0 Hz, 1H), 4.99 (m, 1H), 1.79 (s, 1H), 1.58 (d, *J* = 5.0 Hz, 3H). ^13^C NMR (500 MHz, CDCl_3_, δ ppm): 145.0, 141.0, 140.6, 128.8, 127.3, 125.9, 70.2, 25.1.

## 4. Conclusions

In this study, we investigated the transfer hydrogenation of ketones over electrogenerated base-driven Mn/L catalysts. The results of electrolysis and UV-Vis spectroscopy demonstrate the strong dependence of the catalytic pathway on the solvent used. In i-PrOH, the metal complex catalyst undergoes a similar reaction process to that driven by conventional organic bases, which promotes substrate hydrogenation through the synergistic promotion of the ligand and the metal center to give alcohols with moderate ee values. In contrast, in MeOH, electrochemistry can drive the transfer hydrogenation reaction, which is challenging for conventional organic bases, under mild conditions to obtain racemic alcohols. This study not only elucidates the mechanisms of Mn/L-catalyzed hydrogen transfer reactions in different solvents under electrochemical conditions but also provides valuable experimental and theoretical insights for the development of more efficient and environmentally friendly catalytic processes.

## Figures and Tables

**Table 1:** Electrochemically driven catalysis for asymmetric transfer hydrogenation of acetophenone in i-PrOH.

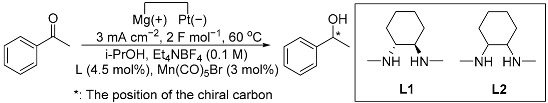				
Entry	Deviation from Standard Conditions	Conversion/%	Selectivity/%	ee/% ^a^
1	None	58	99	63 (R)
2	At 40 °C	50	99	62 (R)
3	At 20 °C	7	98	72 (R)
4	No **L1** and Mn(CO)_5_Br	trace	-	NA.
5	No Mn(CO)_5_Br	trace	-	NA.
6	No **L1**	trace	-	NA.
7	No electricity	trace	-	NA.
8	**L2** instead of **L1**	54	98	NA.
9	PhCOCH_3_, **L1,** and Mn(CO)_5_Br added After electrolysis	57	97	62 (R)
10	t-BuOK (0.2 M), no electricity	61	98	50 (R)

Reaction conditions: undivided cell, Mg anode, Pt cathode, solvent (20 mL), Et_4_NBF_4_ (0.05 M), acetophenone (0.1 M), **L1** (4.5 mol%), Mn(CO)_5_Br (3 mol%), current density = 3 mA cm^−2^. Charge = 2 F mol^−1^. Selectivity = isolate yield/conversion. ^a^ Determined by HPLC with a chiral column.

**Table 2:** Electrochemically driven catalysis for asymmetric transfer hydrogenation of acetophenone in MeOH.

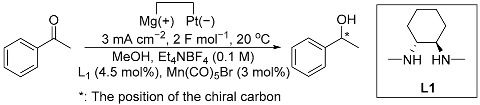				
Entry	Deviation from Standard Conditions	Conversion/%	Selectivity/%	ee/% ^a^
1	None	65	60	NA.
2	at 40 °C	64	64	NA.
3	**L1** (9 mol%) + Mn(CO)_5_Br (6 mol%)	68	79	NA.
4	**L1** (13.5 mol%) + Mn(CO)_5_Br (9 mol%)	71	77	NA.
5	no electricity	-	-	NA.
6	t-BuOK (0.2 M), no electricity	Trace	-	NA.

Reaction conditions: undivided cell, Mg anode, Pt cathode, solvent (20 mL), Et_4_NBF_4_ (0.05 M), acetophenone (0.1 M), **L1** (4.5 mol%), Mn(CO)_5_Br (3 mol%), current density = 3 mA cm^−2^. Charge = 2 F mol^−1^. Selectivity = isolate yield/conversion. ^a^ Determined by HPLC with a chiral column.

## Data Availability

The data that support the findings of this study are available in the Supplementary Materials of this article.

## Supplementary Materials

The following supporting information can be downloaded at: https://www.mdpi.com/article/10.3390/molecules30040910/s1, Table S1: The conditions for unsaturated bond transfer hydrogenation reactions using methanol as a hydrogen source; Figure S1: CV curves recorded in DMF containing 0.05 M Et_4_NBF_4_: (a) blank solution, (b) as a + 1.125 mol% R,R-N1,N2-dimethylcyclohexane-1,2-diamine, (c) as a + 0.75 mol% MnCO_5_Br, (d) b + 0.75 mol% MnCO_5_Br; Figure S2: CV curves recorded in i-PrOH containing 0.05 M Et_4_NBF_4_: (a) blank solution, (b) as a + 10 mM acetophenone; Figure S3: CV curves recorded in MeOH containing 0.05 M Et_4_NBF_4_: (a) blank solution, (b) as a + 10 mM acetophenone; Figure S4: UV-Vis absorption spectra of (a) MnCO_5_Br/L1(1/1.5) in i-PrOH containing 0.05 M Et_4_NBF_4_, (b) as a + t-BuOK (0.05 M); Figure S5: The ^1^H NMR spectrum of deuterated-1-phenylethanol; Figures S6–S23: ^1^H NMR and ^13^C NMR spectra of compounds of 1-Phenethyl alcohol, 1-(p-tolyl)ethanol, 1-(m-tolyl)ethanol, 2-methyl-1-phenylpropan-1-ol, 1-(4-(trifluoromethyl)phenyl)ethanol, 1-(4-chlorophenyl)ethanol, 1-(4-bromophenyl)ethanol, 1-(naphthalen-2-yl)ethanol, 1-([1,1′-biphenyl]-4-yl)ethanol.
