# Supplementary material for: Effect of Solvents on Electrogenerated Base-Driven Transfer Hydrogenation Reactions
Source: Molecules. 2025 Feb 15;30(4):910. doi: 10.3390/molecules30040910 (PMC11858110; doi:10.3390/molecules30040910)
Supplement: Supplementary file 1 [file molecules-30-00910-s001.zip › molecules-3451735-supplementary.pdf]

General information

$$ee = \frac{[R] - [S]}{[R] + [S]} \times 100\%$$

Equation S1

Table S1 The conditions for unsaturated bond transfer hydrogenation reactions using methanol as a hydrogen source[a].

Entry	Base	Catalyst type[b]	Temperature (°C)	Substrate	Yield (%)	Ref.
1	EGB	Mn/L1	20	aromatic ketones	78	This work
2	$KHCO_3$	Ru/L2	110	ketones	84	[1]
3	K_2CO_3	Rh/L3	90	α,β-unsaturated ketones	97	[2]
4	$CsCO_3$	Co/L4	140	α,β-unsaturated ketones	91	[3]

[a]Due to the limited cases of methanol as a hydrogen source for the transfer hydrogenation of aromatic ketones, we have included additional literature on the transfer hydrogenation of unsaturated bonds using methanol as a hydrogen source for comparison. [b]L1 ((1R,2R)-N1,N2-dimethylcyclohexane-1,2-diamine), L2 (N-benzyl-2,6-di(1H-imidazol-1-yl)-N-methylpyridin-4-amine), L3 (4-(1-((4-methoxyphenyl)imino)ethyl)phenol), L4 (1,10-phenanthroline),

Computational Details

Density functional theory (DFT) calculations were carried out using Gaussian 16 programs [4] throughout this manuscript. Geometric optimizations of the reactants, transition states, and products were performed using M06-L functional [5]. The standard 6-311G(d,p) basis set [6-8] for H, C, N and O was used. For the Mn and Br atoms, the SDD basis set and its corresponding effective core potential [9-10] was used. Harmonic vibration frequency calculations were performed for all stationary points to confirm them as a local minima or transition state. The intrinsic reaction coordinate (IRC) scheme [11-12] was applied for the calculations of the reaction coordinates to confirm whether or not the transition states were directly connected to the reactants and products. Approximate solvent effects (solvent = MeOH) were taken into consideration based on the SMD continuum solvation model [13] in all calculations.

Figures of Cyclic Voltammetry

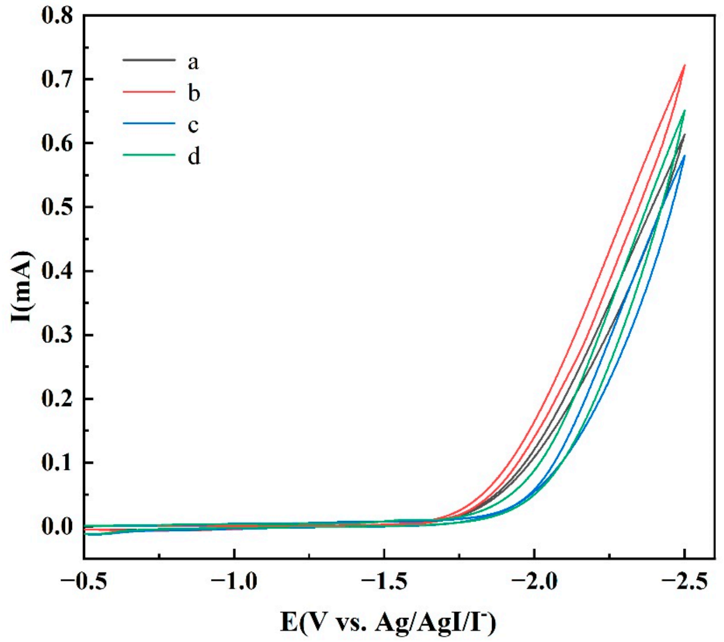

Figure S1 CV curves recorded in DMF containing 0.05 M Et_4NBF_4: (a) blank solution, (b) as a + 1.125 mol% R,R-N1,N2-dimethylcyclohexane-1,2-diamine, (c) as a + 0.75 mol% $Mn(CO)_5Br$, (d) b + 0.75 mol% $Mn(CO)_5Br$

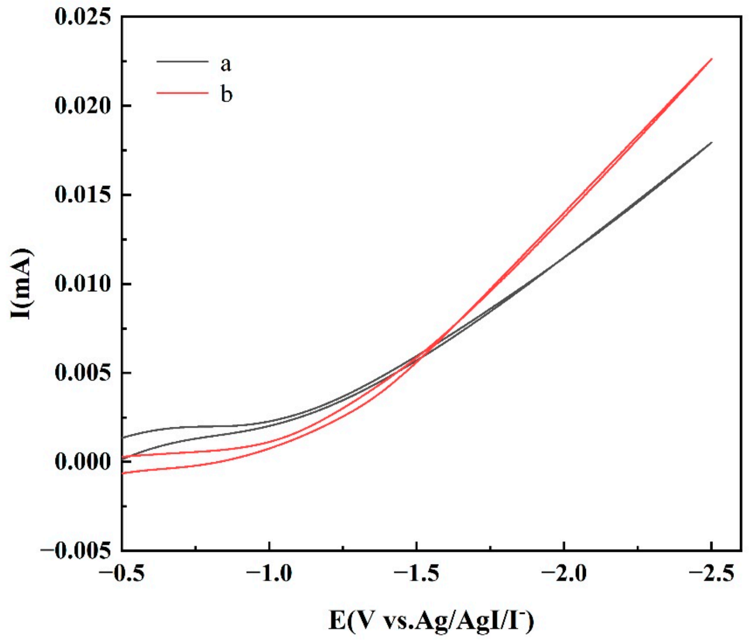

Figure S2 CV curves recorded in i-PrOH containing 0.05 M Et_4NBF_4: (a) blank solution, (b) as a + 10 mM acetophenone.

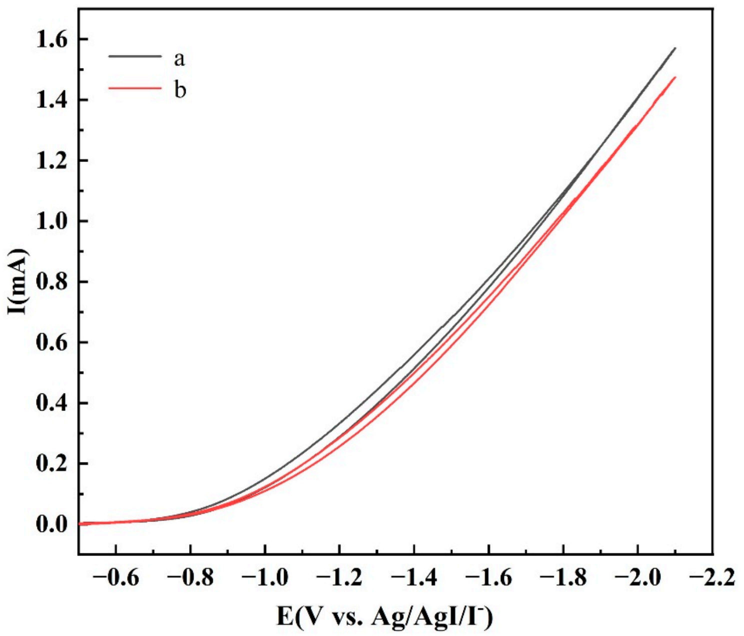

Figure S3 CV curves recorded in MeOH containing 0.05 M Et_4NBF_4: (a) blank solution, (b) as a + 10 mM acetophenone.

Figures of UV-Vis absorption spectrum

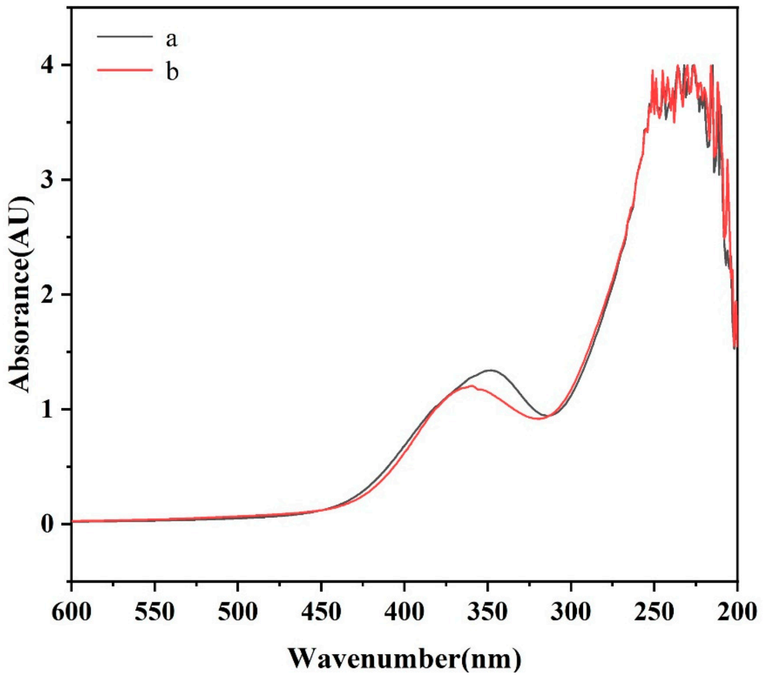

Figure S4 UV-Vis absorption spectra of (a) $Mn(CO)_5Br$/L1(1/1.5) in i-PrOH containing 0.05 M Et_4NBF_4, (b) as a + t-BuOK (0.05 M).

NMR spectra

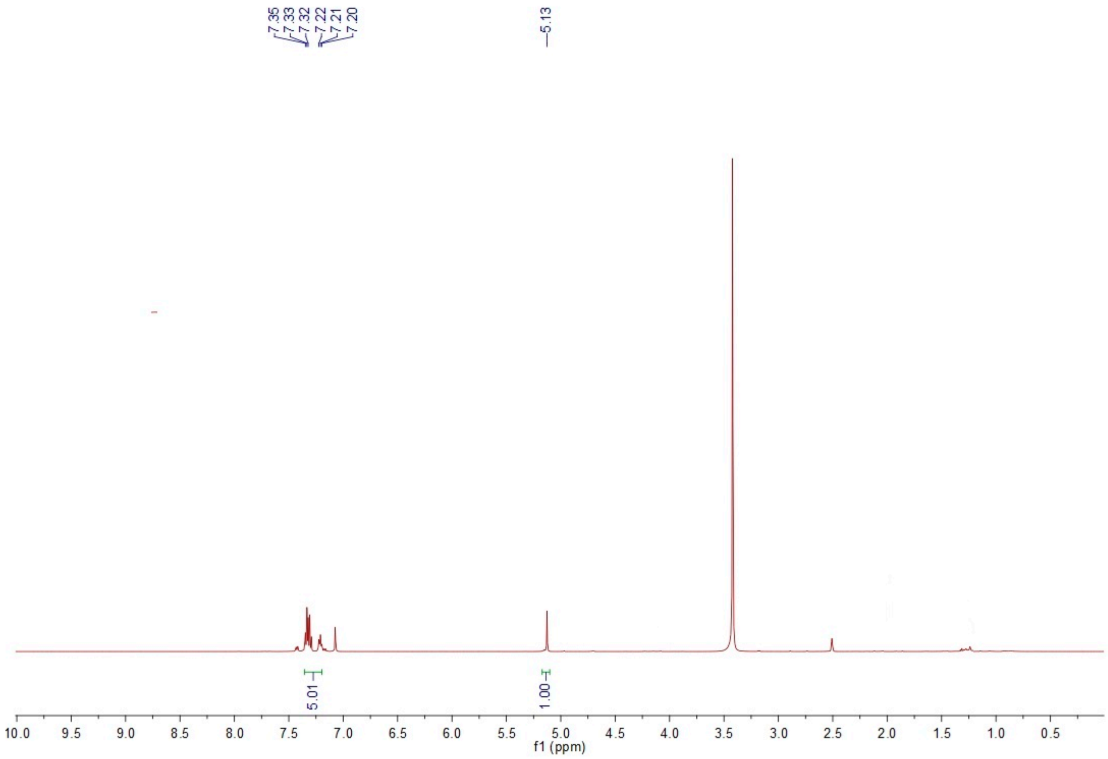

Figure S5 The ^{1}H NMR spectrum of deuterated-1-phenylethanol in DMSO-*d6*

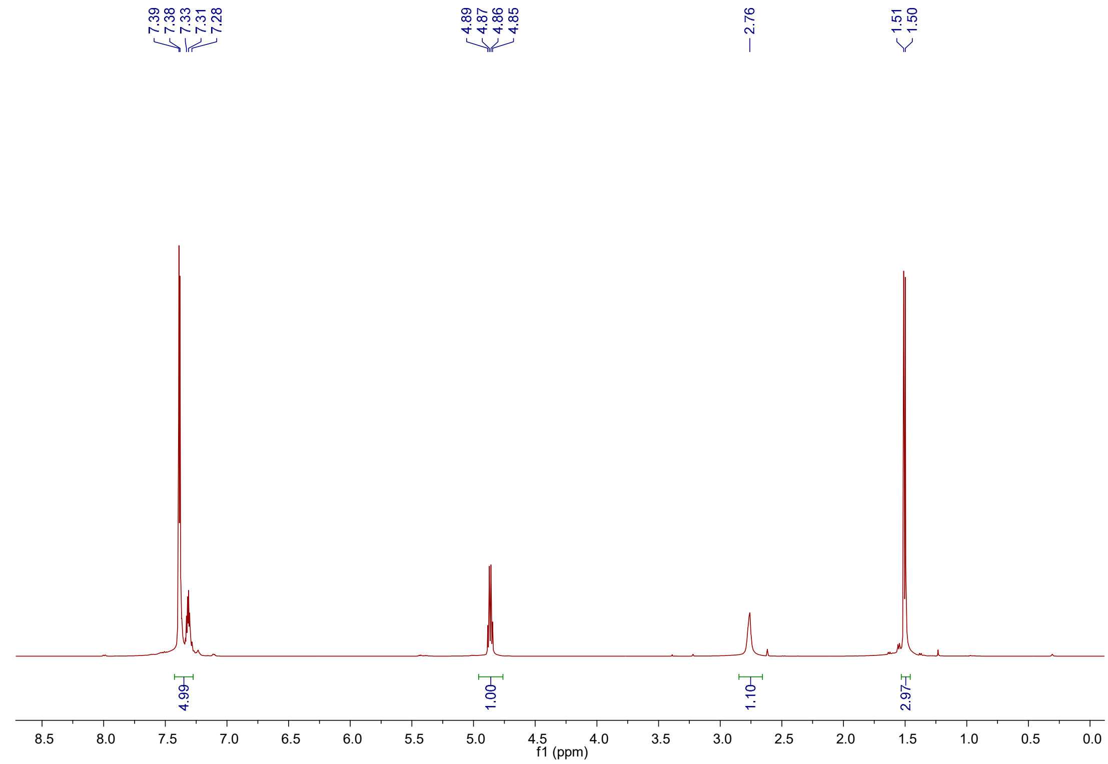

Figure S6 The ^{1}H NMR spectrum of 1-phenylethanol in $CDCl_3$

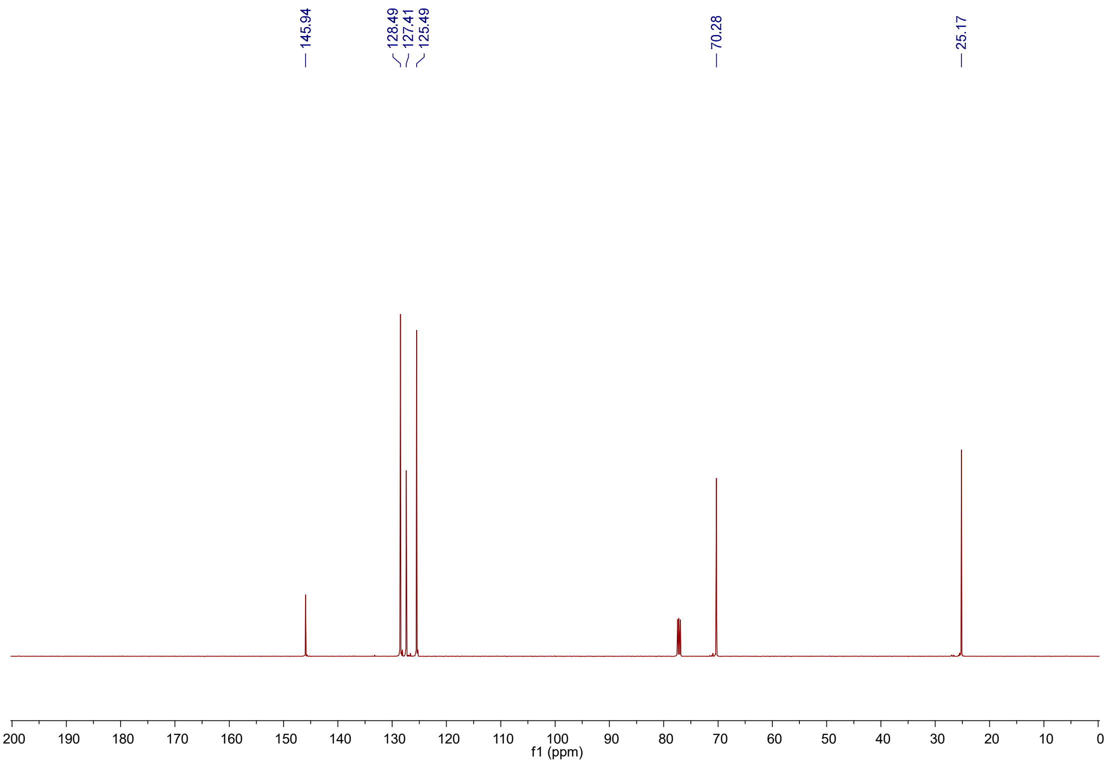

Figure S7 The ^{13}C NMR spectrum of 1-phenylethanol in $CDCl_3$

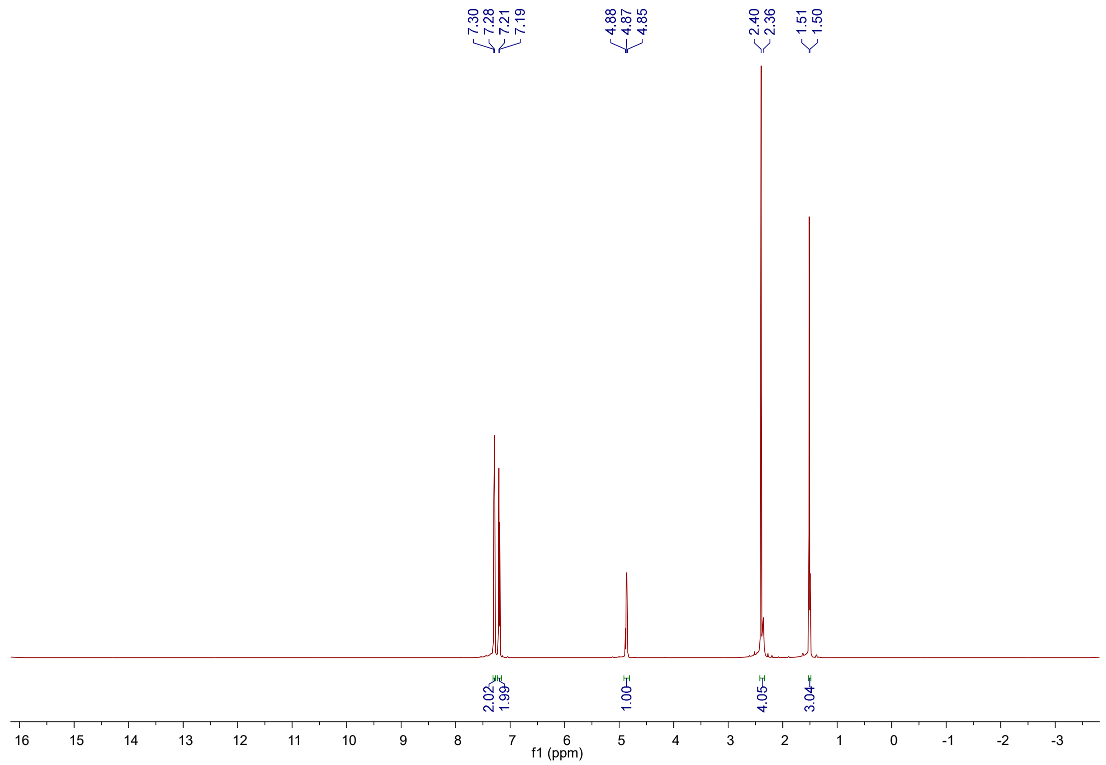

Figure S8 The ^{1}H NMR spectrum of 1-(p-tolyl)ethanol in $CDCl_3$

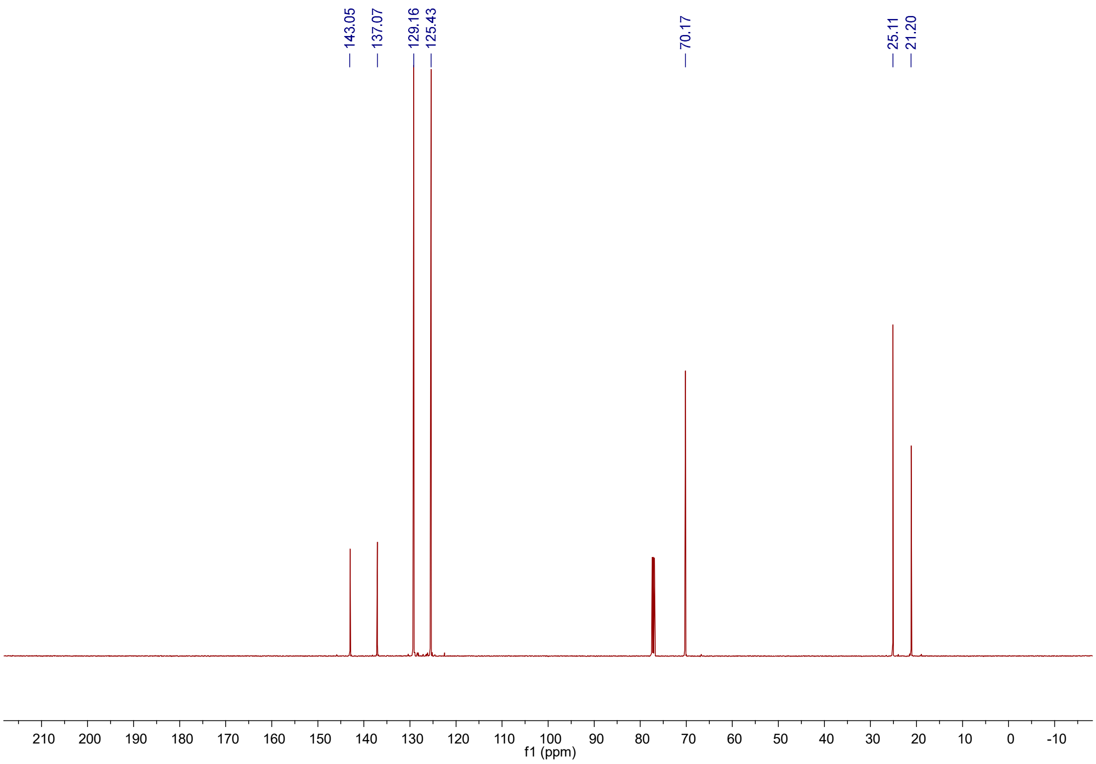

Figure S9 The ^{13}C NMR spectrum of 1-(p-tolyl)ethanol in $CDCl_3$

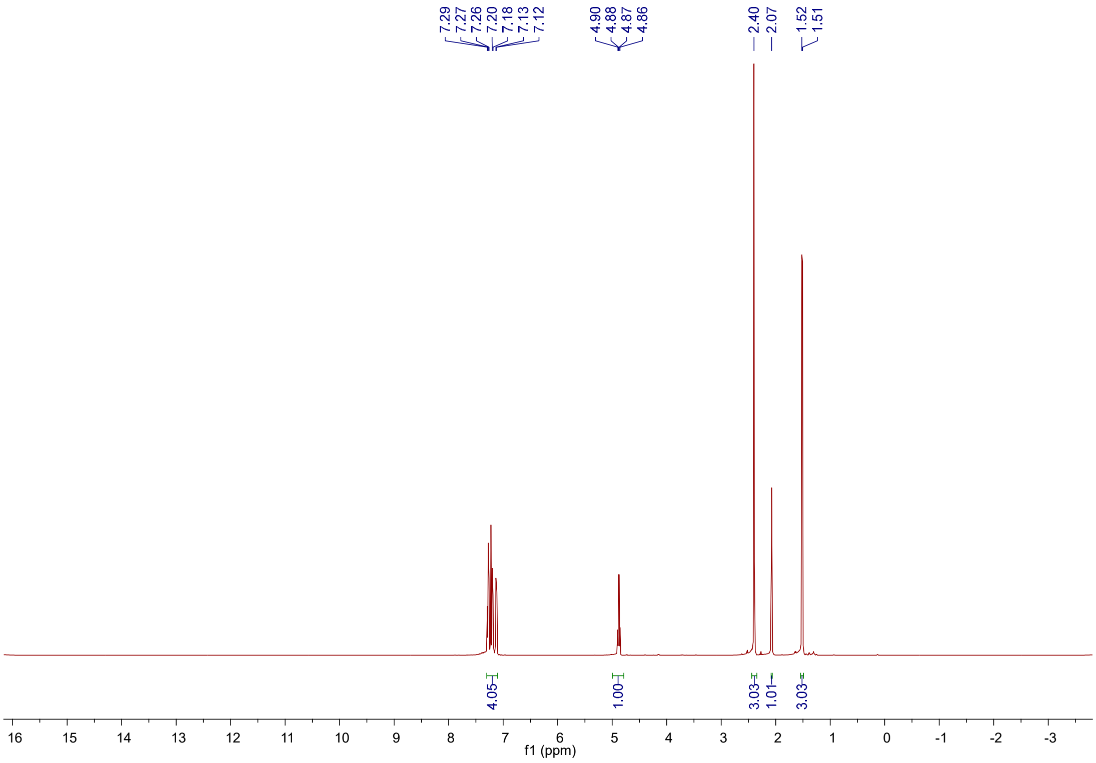

Figure S10 The ^{1}H NMR spectrum of 1-(m-tolyl)ethanol in $CDCl_3$

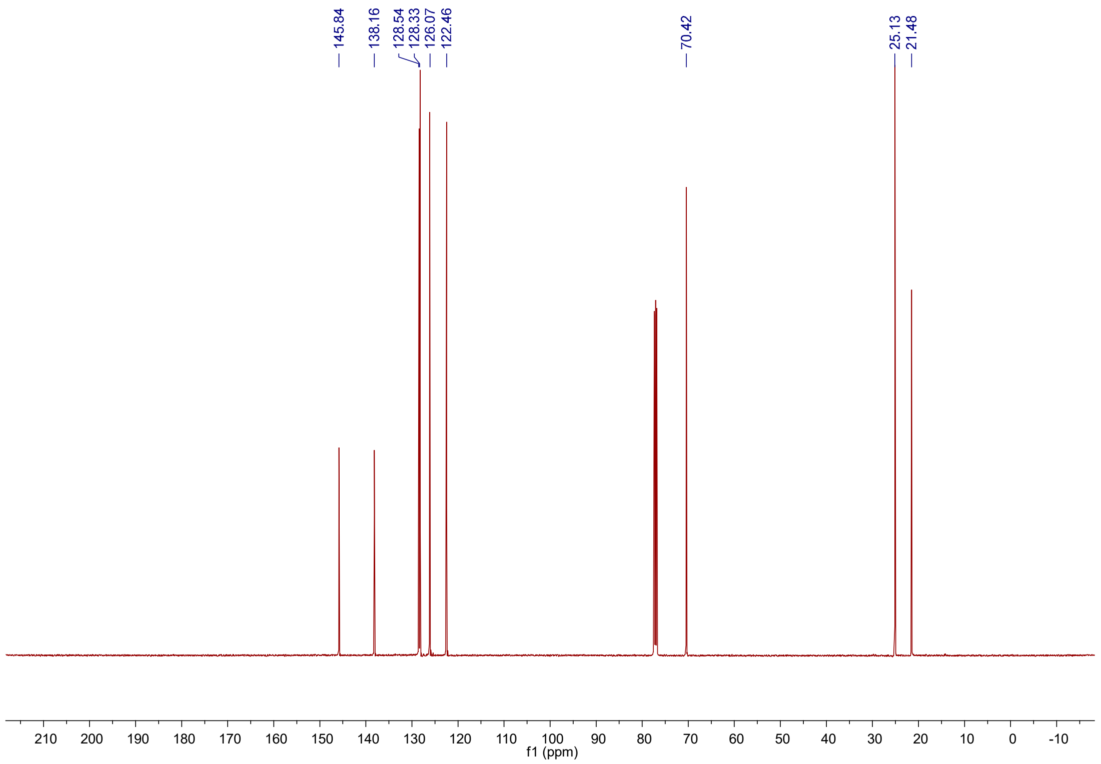

Figure S11 The ^{13}C NMR spectrum of 1-(m-tolyl)ethanol in $CDCl_3$

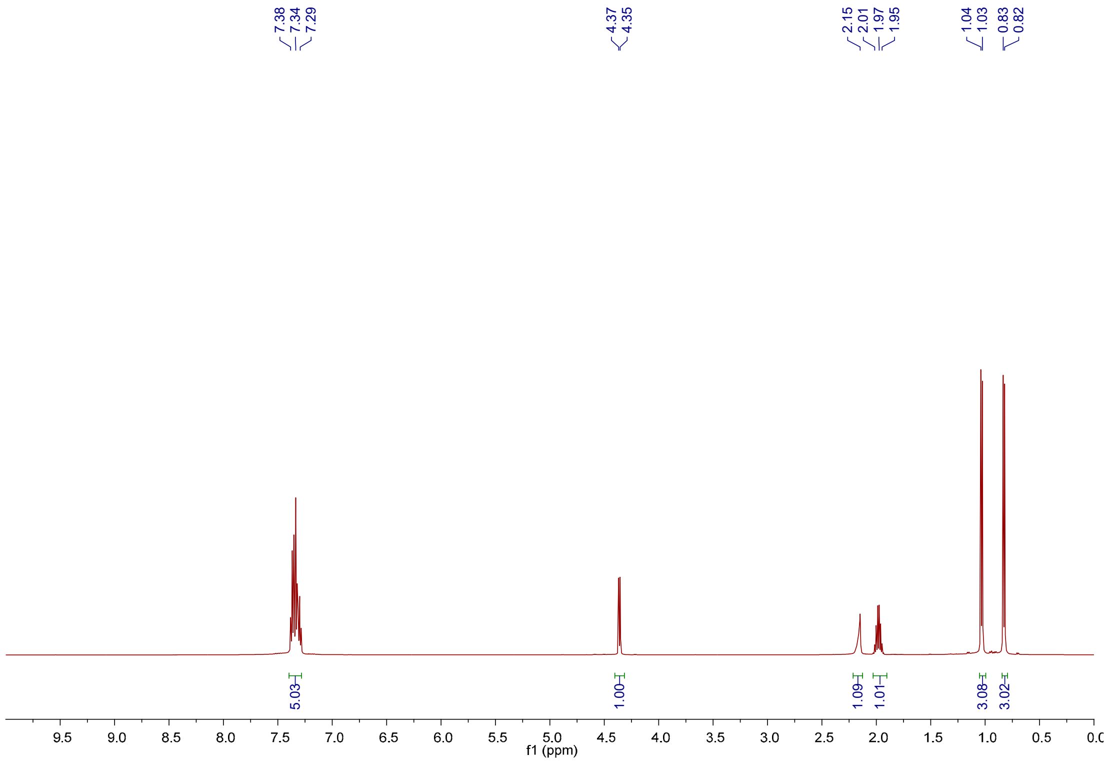

Figure S12 The ^{1}H NMR spectrum of 2-methyl-1-phenylpropan-1-ol in $CDCl_3$

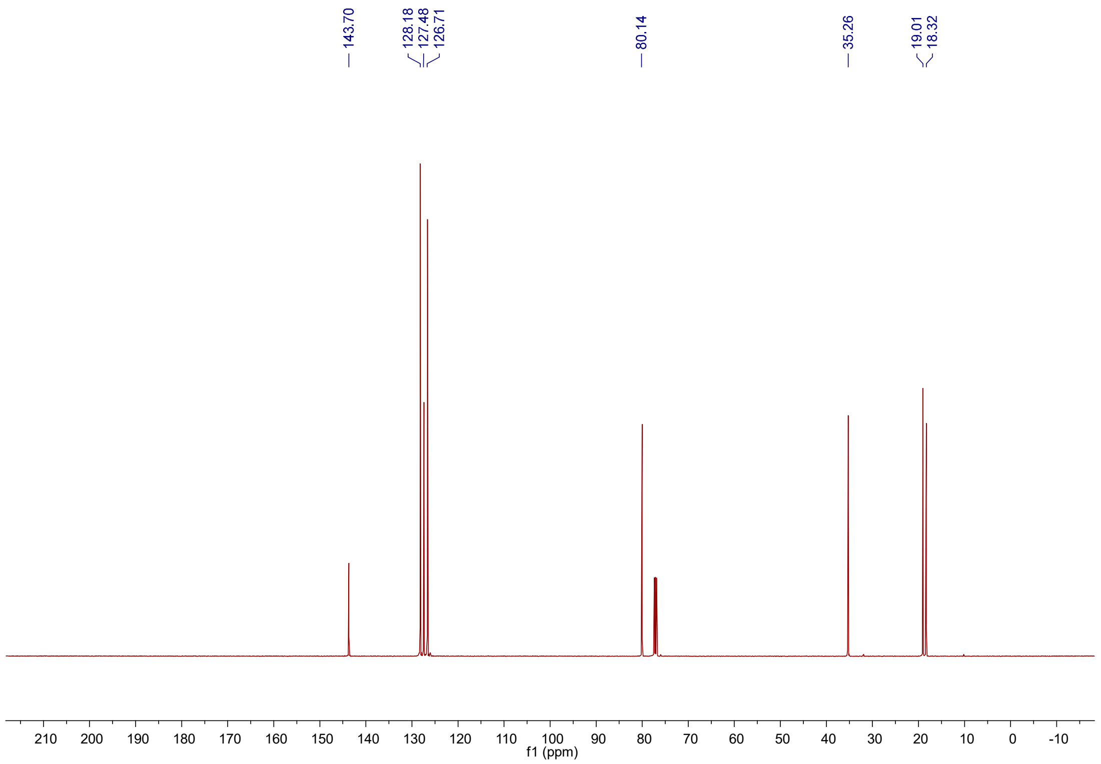

Figure S13 The ^{13}C NMR spectrum of 2-methyl-1-phenylpropan-1-ol in $CDCl_3$

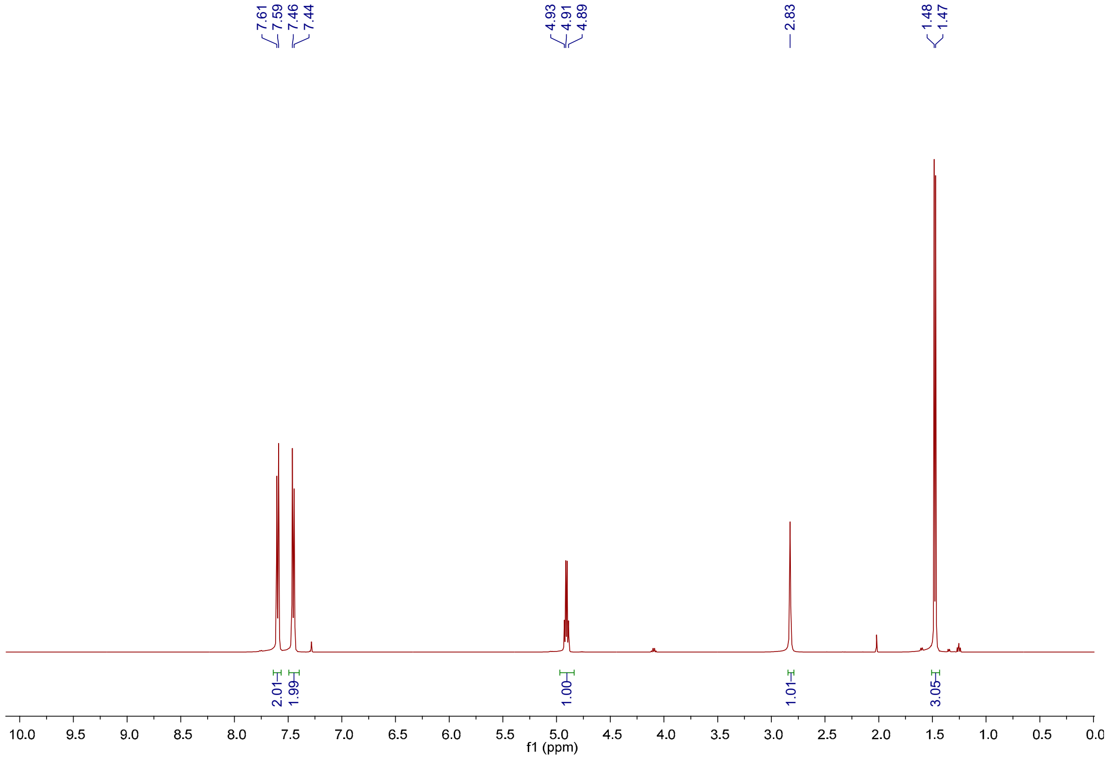

Figure S14 The ^{1}H NMR spectrum of 1-(4-(trifluoromethyl)phenyl)ethanol in $CDCl_3$

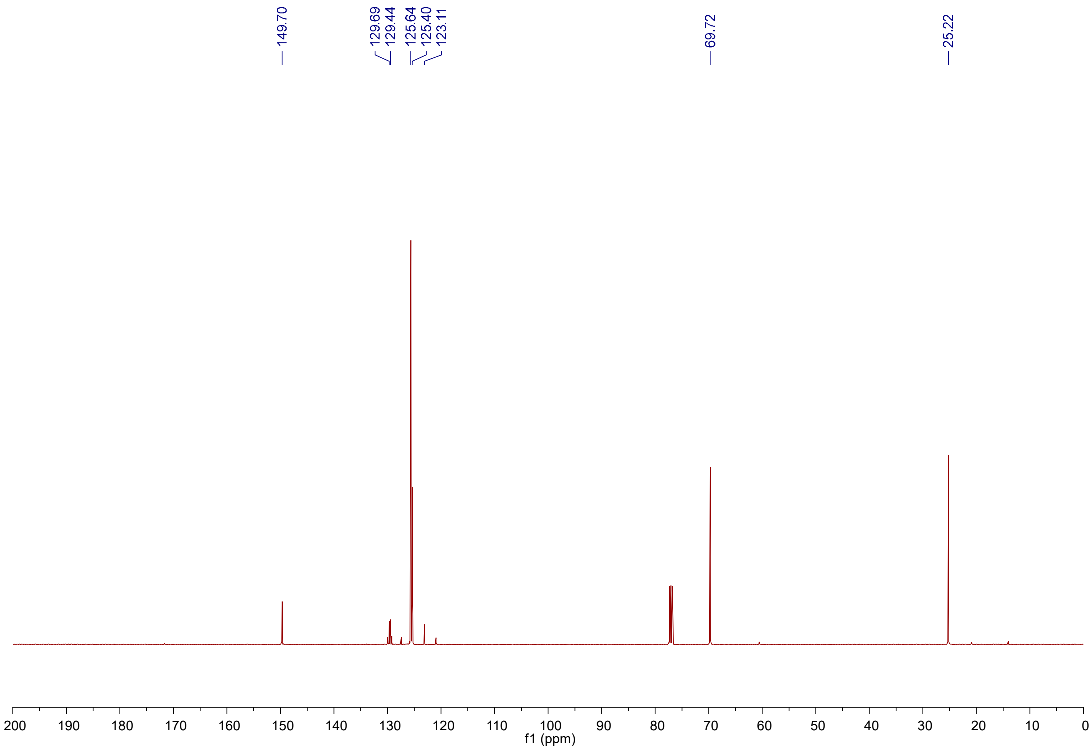

Figure S15 The ^{13}C NMR spectrum of 1-(4-(trifluoromethyl)phenyl)ethanol in $CDCl_3$

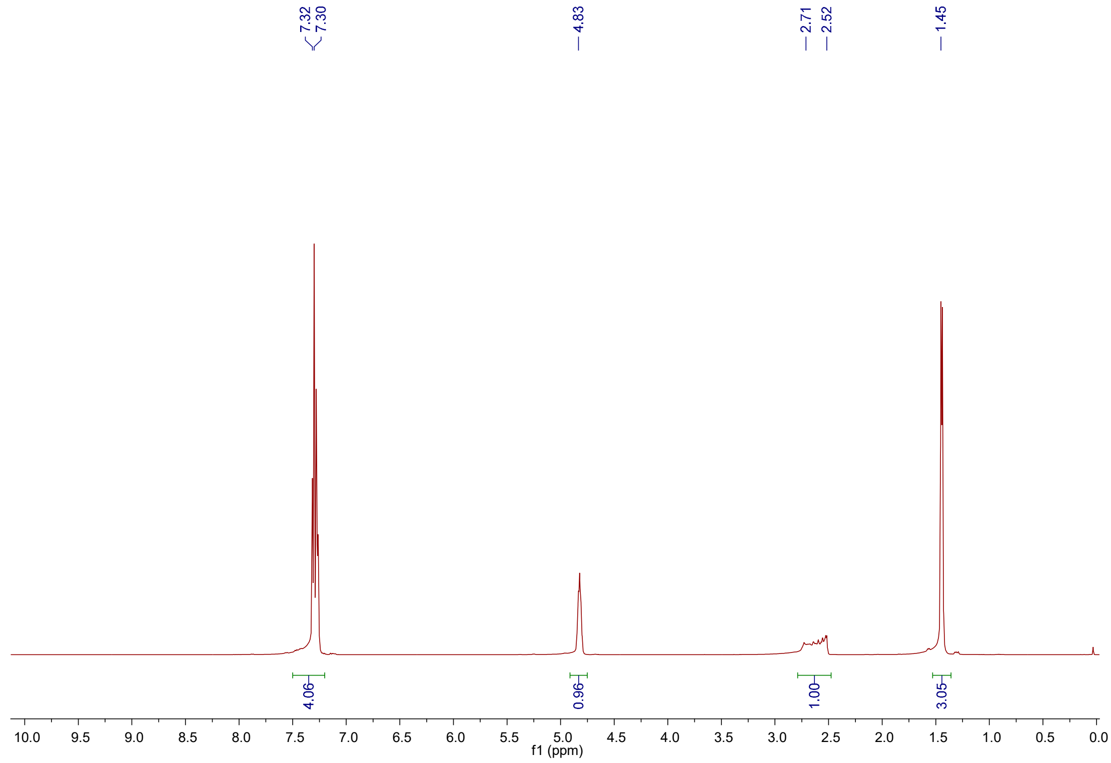

Figure S16 The ^{1}H NMR spectrum of 1-(4-chlorophenyl)ethanol in $CDCl_3$

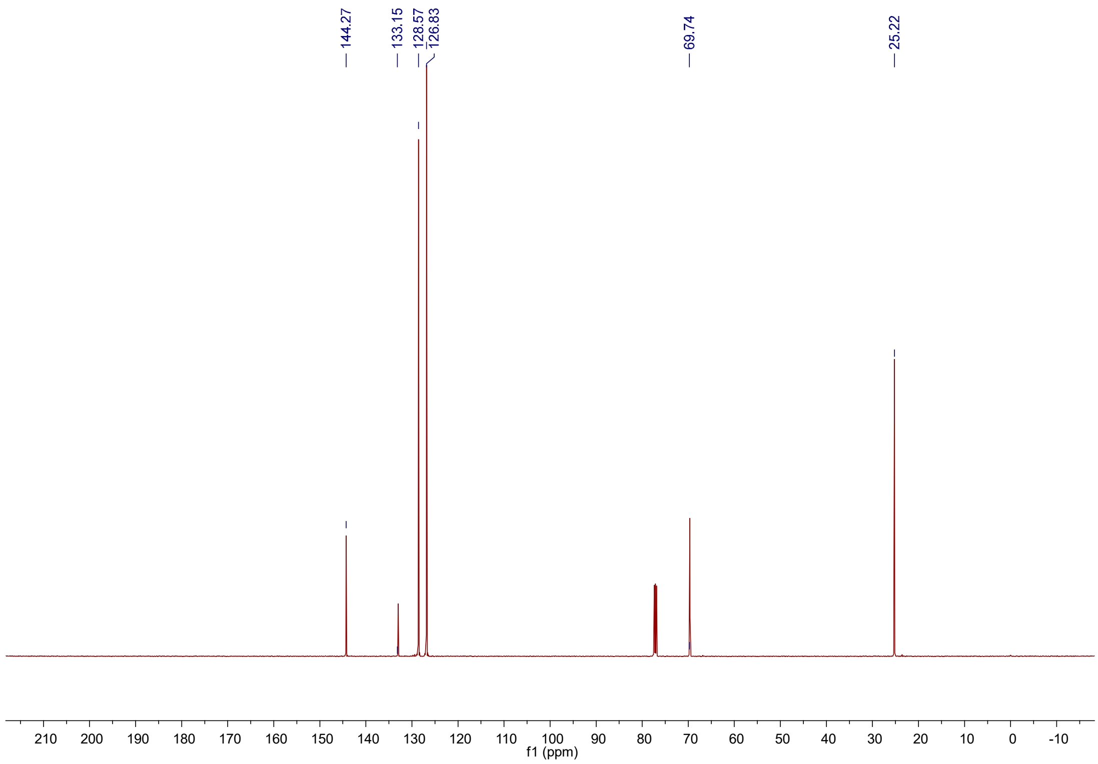

Figure S17 The ^{13}C NMR spectrum of 1-(4-chlorophenyl)ethanol in $CDCl_3$

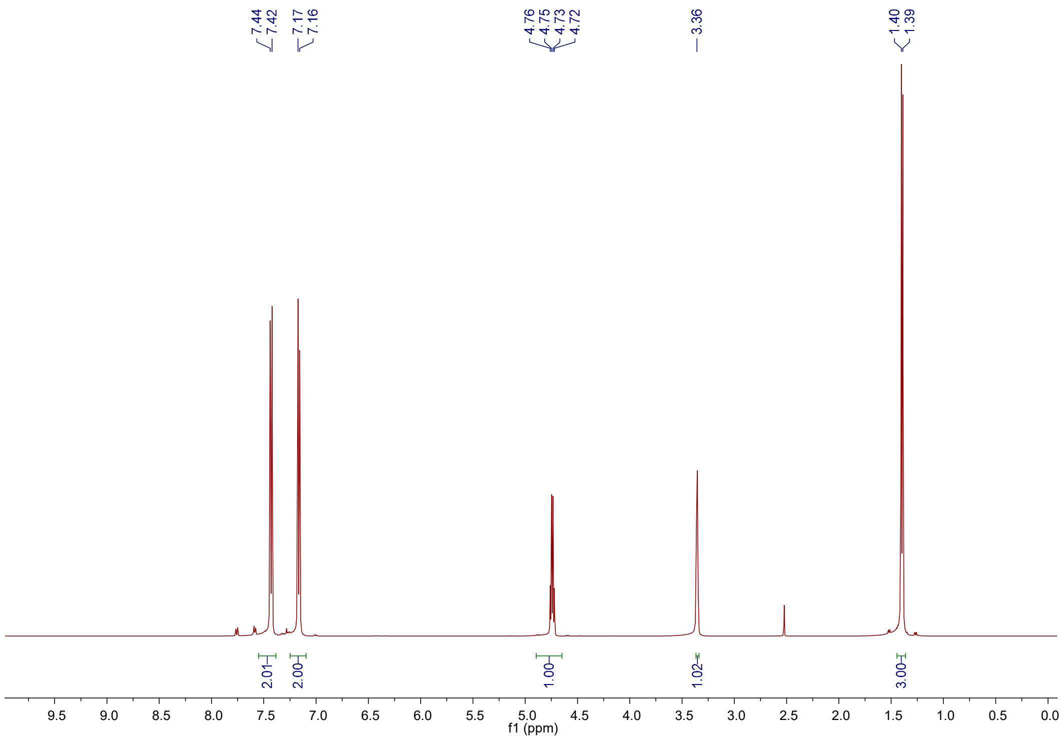

Figure S18 The ^{1}H NMR spectrum of 1-(4-bromophenyl)ethanol in $CDCl_3$

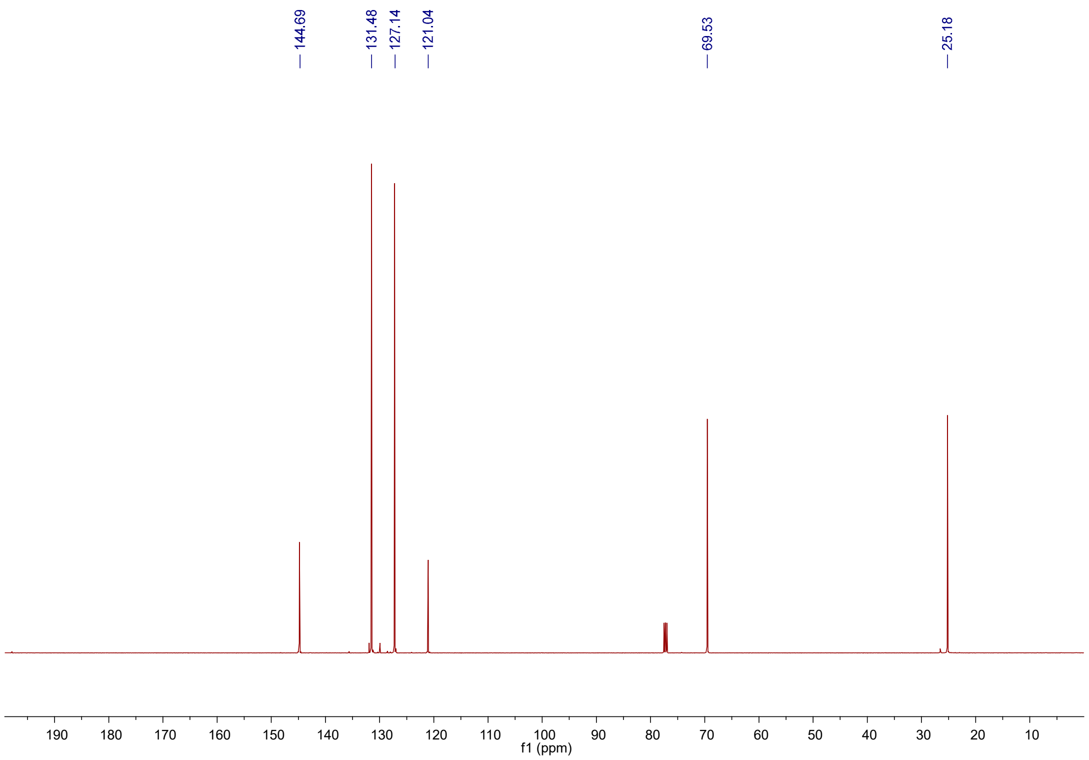

Figure S19 The ^{13}C NMR spectrum of 1-(4-bromophenyl)ethanol in $CDCl_3$

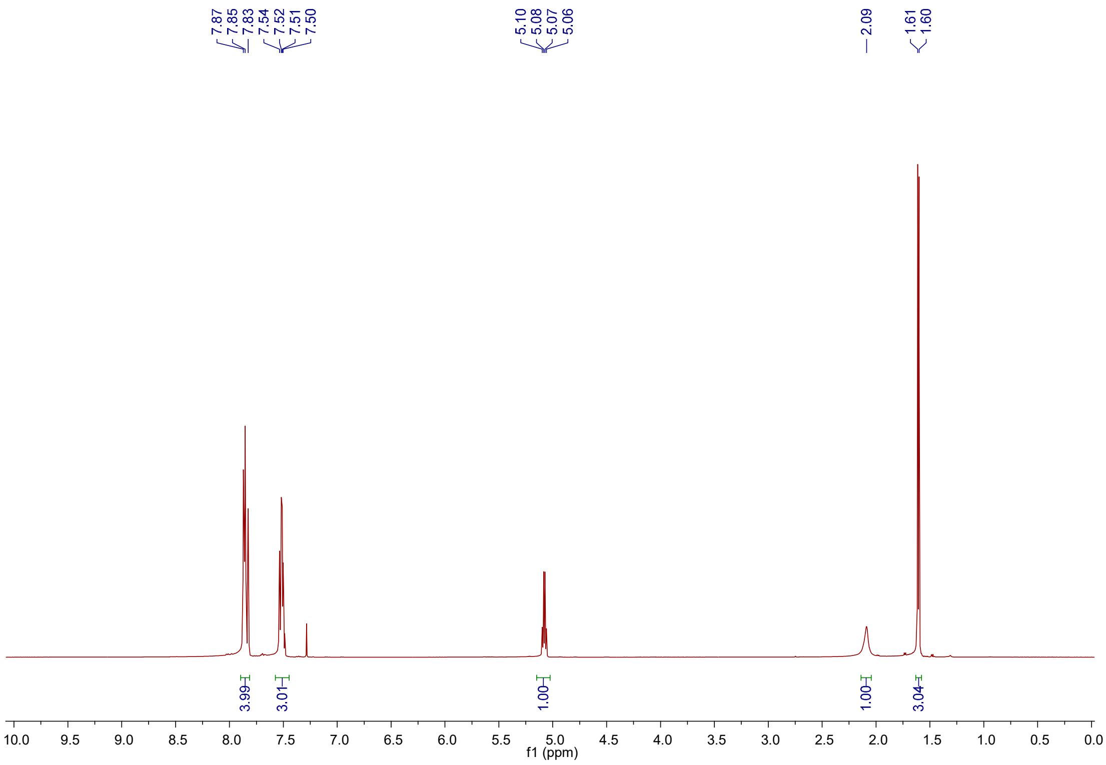

Figure S20 The ^{1}H NMR spectrum of 1-(naphthalen-2-yl)ethanol in $CDCl_3$

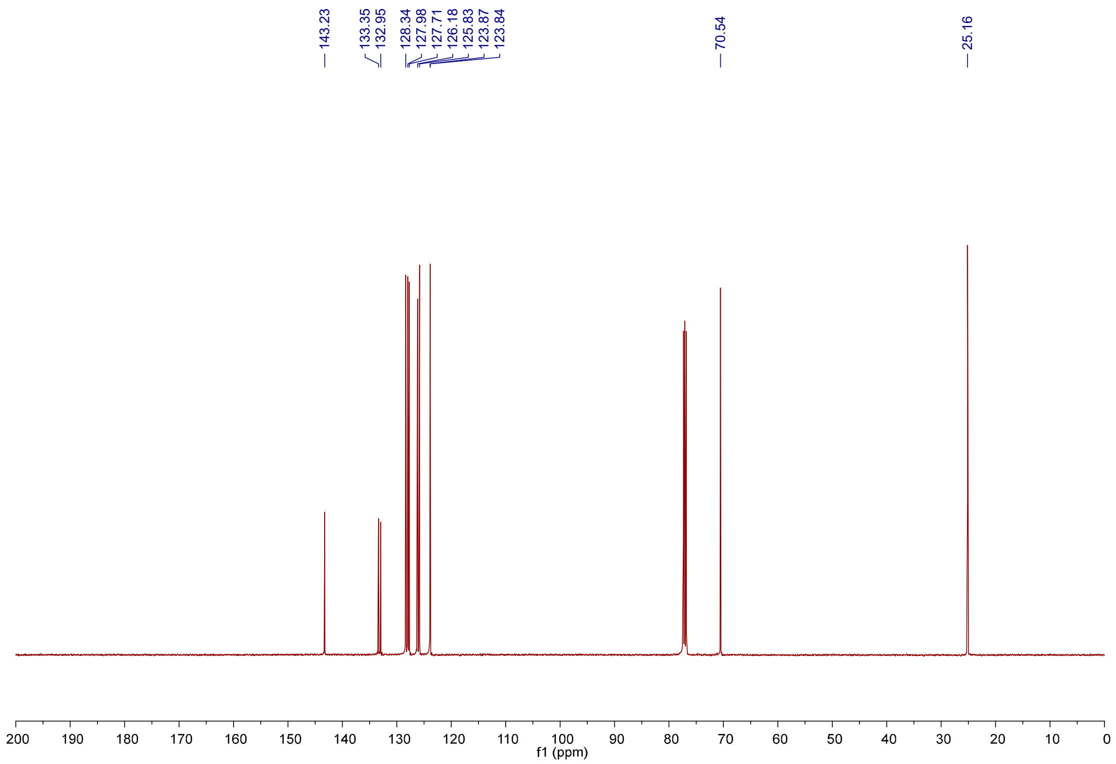

Figure S21 The ^{13}C NMR spectrum of 1-(naphthalen-2-yl)ethanol in $CDCl_3$

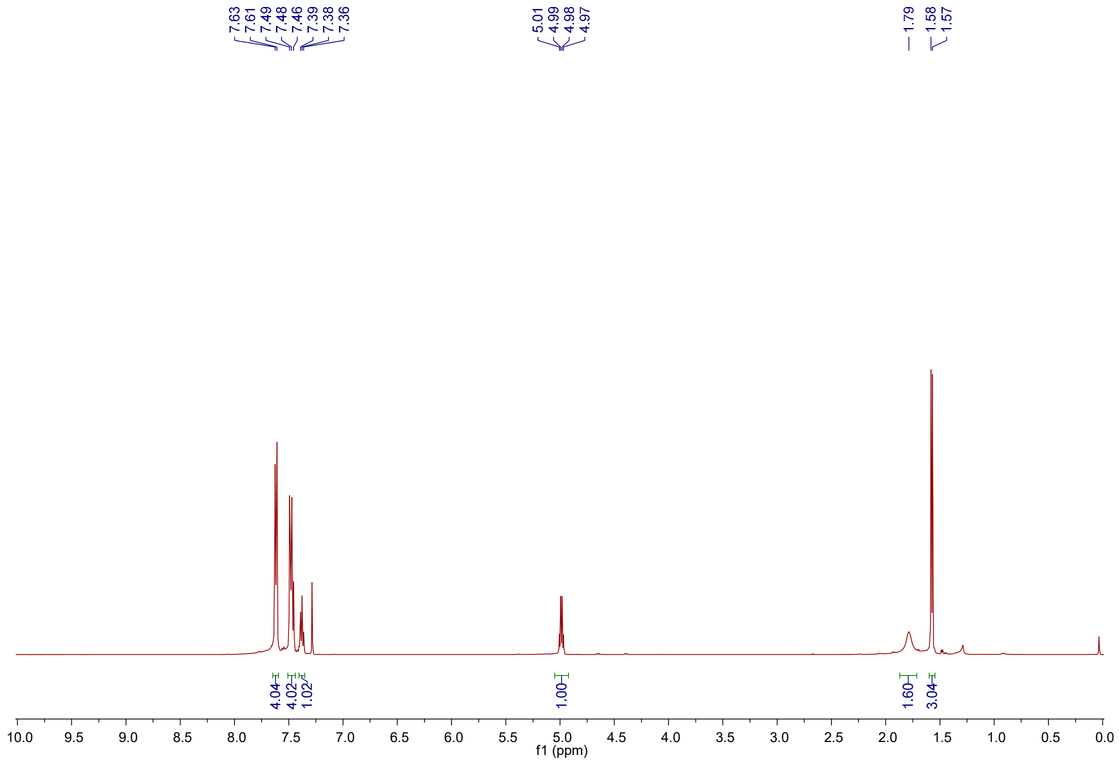

Figure S22 The ^{1}H NMR spectrum of 1-([1,1'-biphenyl]-4-yl)ethanol in $CDCl_3$

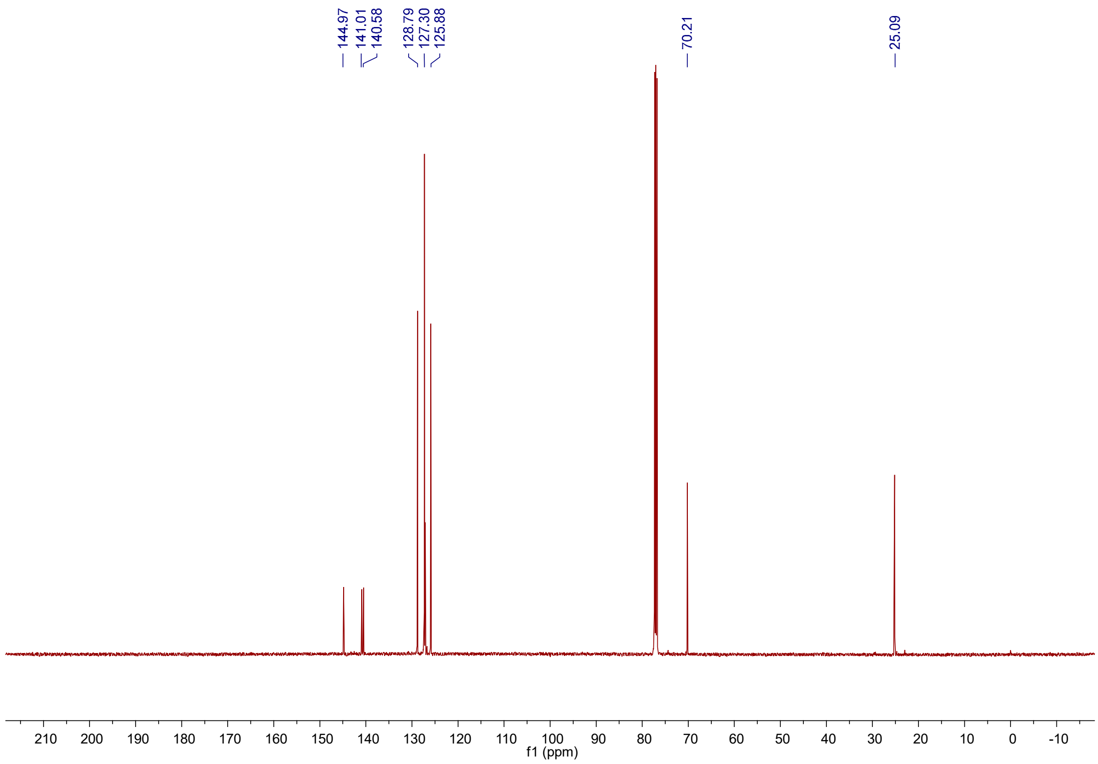

Figure S23 The ^{13}C NMR spectrum of 1-([1,1'-biphenyl]-4-yl)ethanol in $CDCl_3$

Chromatograms of compounds 1a-1i

1-phenylethanol

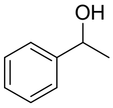

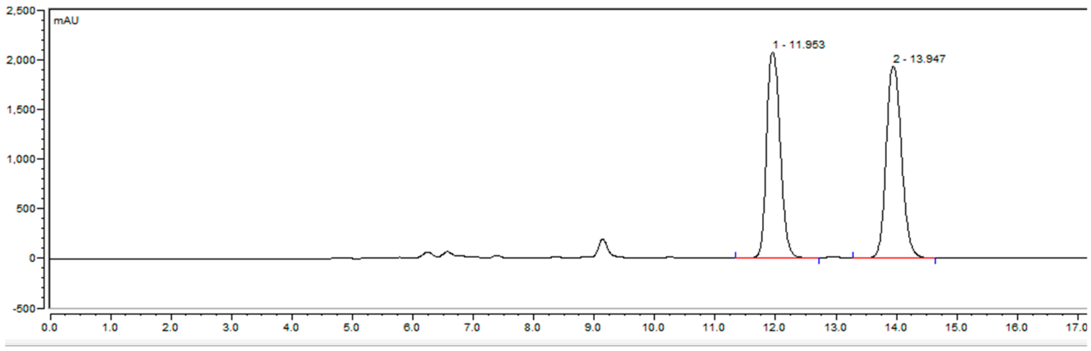

Peak#	Ret. time	Area	Height	Area%
1	11.953	539.8066	2072.435	48.76
2	13.947	567.1485	1931.796	51.24

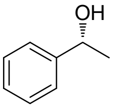

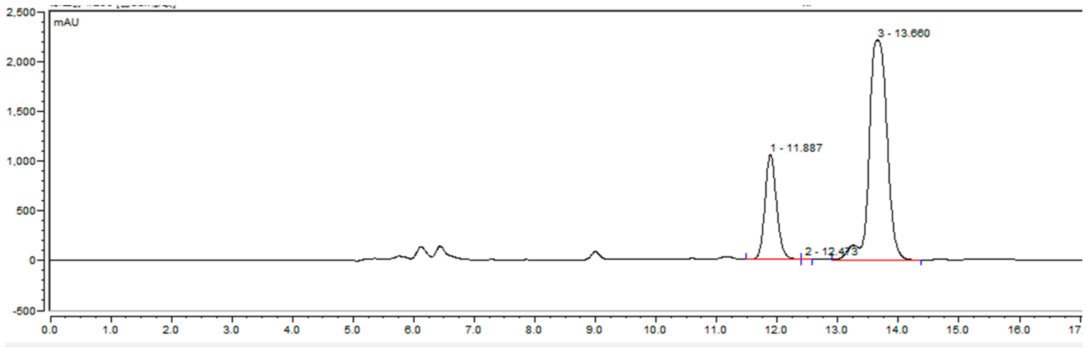

Peak#	Ret.time	Area	Height	Area%
1	11.907	189.8494	912.120	21.23
2	13.667	704.6718	2141.235	78.77

1-(p-tolyl)ethanol

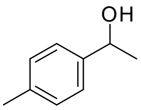

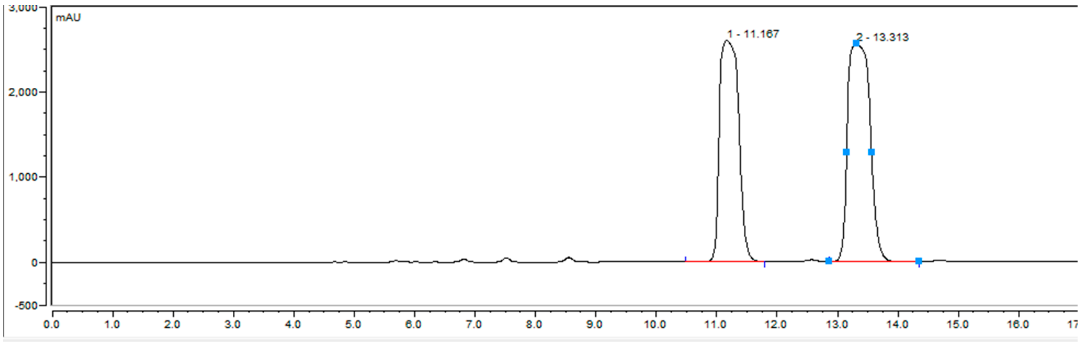

Peak#	Ret.time	Area	Height	Area%
1	11.167	998.3361	2596.555	48.81
2	13.313	1046.1639	2557.257	51.19

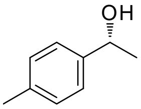

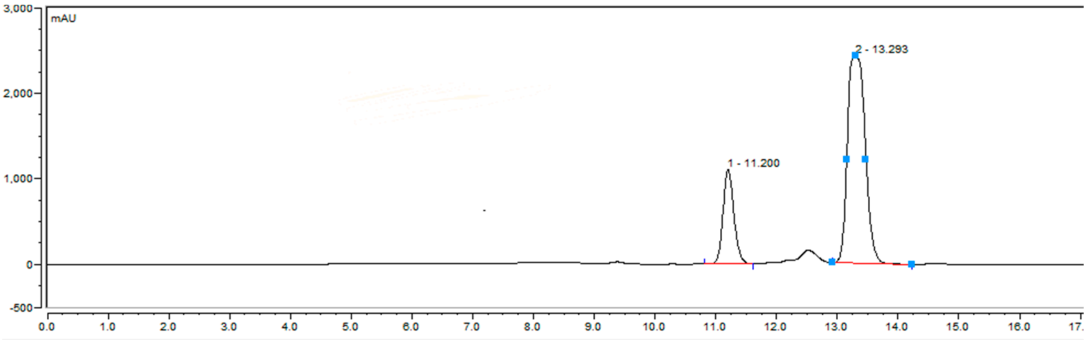

Peak#	Ret.time	Area	Height	Area%
1	11.200	250.8193	1100.446	24.00
2	13.293	794.2611	2424.091	76.00

1-(m-tolyl)ethanol

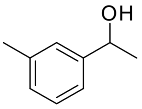

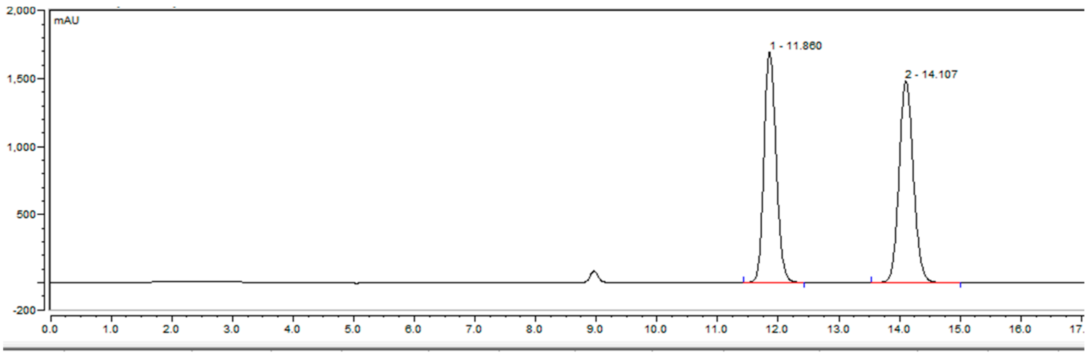

Peak#	Ret.time	Area	Height	Area%
1	11.860	392.4690	1692.957	49.24
2	14.107	404.6603	1481.950	50.76

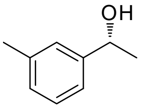

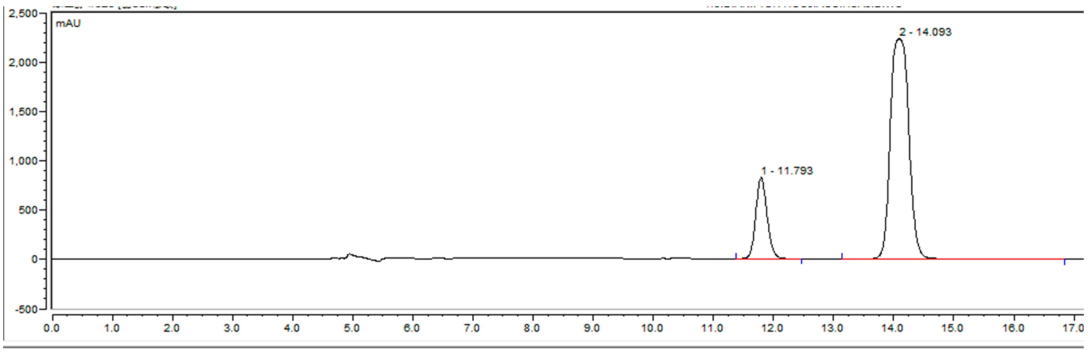

Peak#	Ret.time	Area	Height	Area%
1	11.793	193.7080	821.677	19.90
2	14.093	779.8319	2230.308	80.10

2-methyl-1-phenylpropan-1-ol

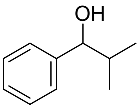

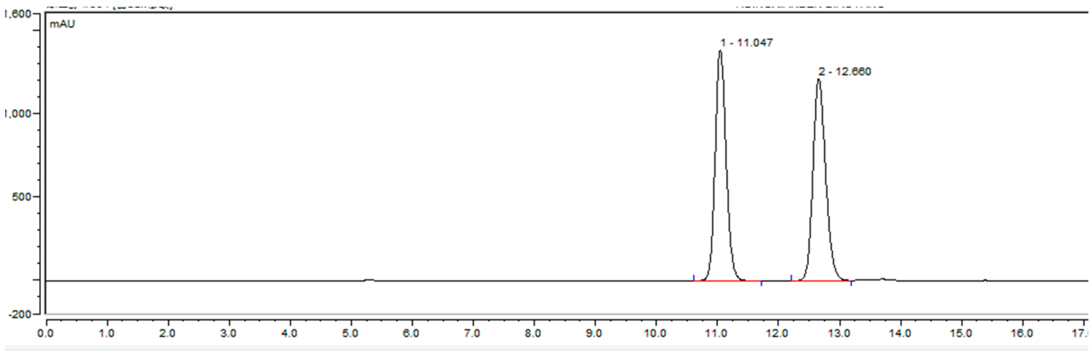

Peak#	Ret.time	Area	Height	Area%
1	11.047	286.0990	1383.298	49.60
2	12.660	290.6900	1209.071	50.40

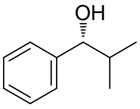

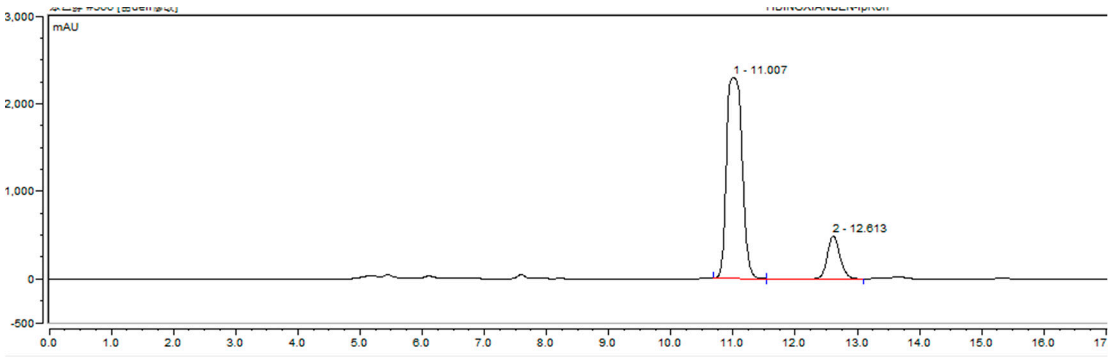

Peak#	Ret.time	Area	Height	Area%
1	11.007	665.6240	2291.482	85.26
2	12.613	115.0727	490.219	14.74

1-(4-(trifluoromethyl)phenyl)ethanol

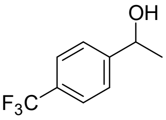

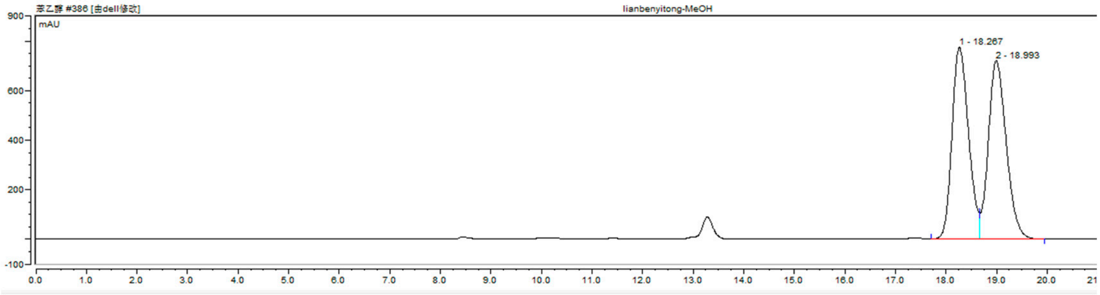

Peak#	Ret.time	Area	Height	Area%
1	18.227	607.1607	1545.278	48.87
2	18.940	635.1292	1446.120	51.13

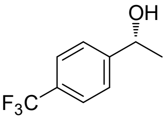

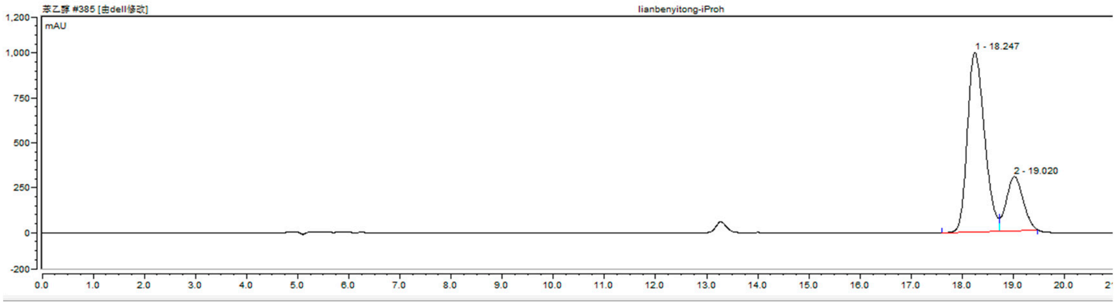

Peak#	Ret.time	Area	Height	Area%
1	18.247	390.6704	1001.054	75.97
2	19.020	123.5626	311.203	24.03

1-(4-chlorophenyl)ethanol

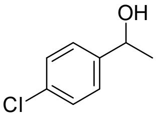

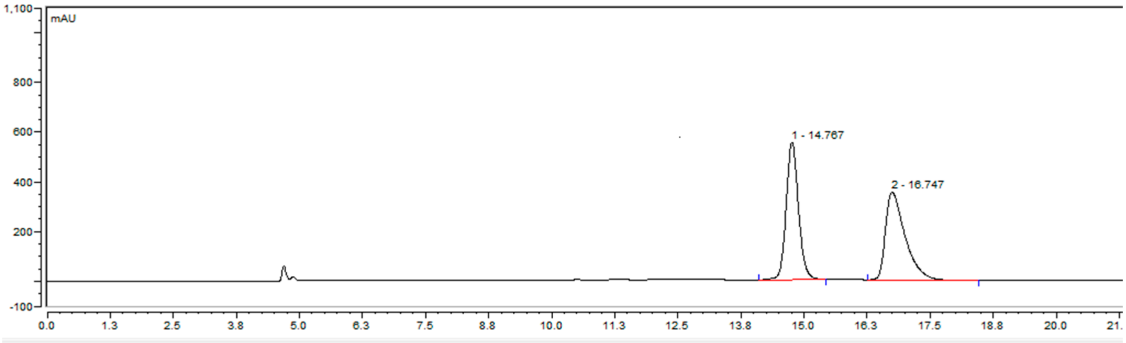

Peak#	Ret.time	Area	Height	Area%
1	14.767	156.0660	551.364	49.52
2	16.747	159.0971	353.063	50.48

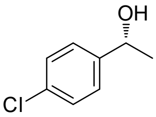

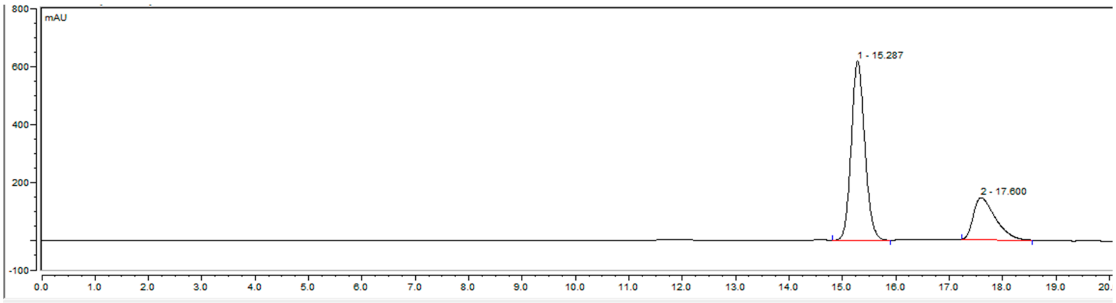

Peak#	Ret.time	Area	Height	Area%
1	15.287	181.3429	616.256	72.90
2	17.600	67.4101	143.772	27.10

1-(4-bromophenyl)ethanol

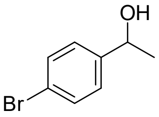

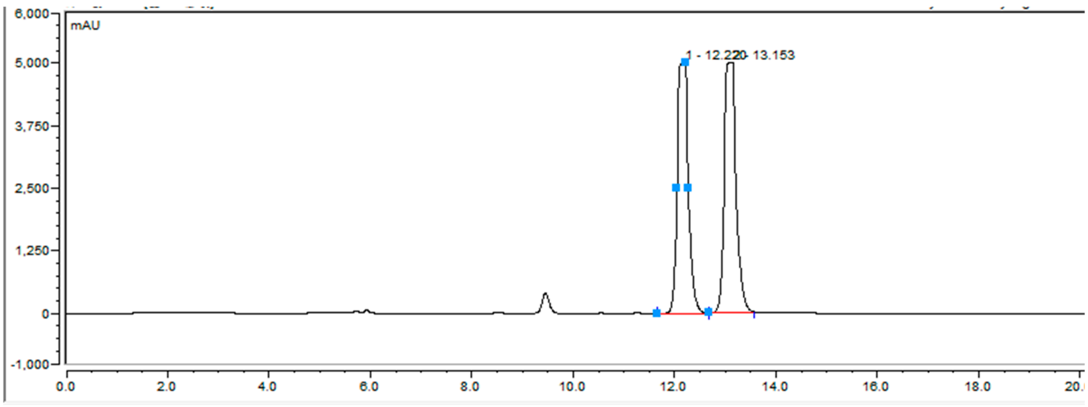

Peak#	Ret.time	Area	Height	Area%
1	12.220	1266.7912	4990.342	48.79
2	13.153	1329.8152	4974.680	51.21

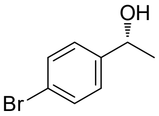

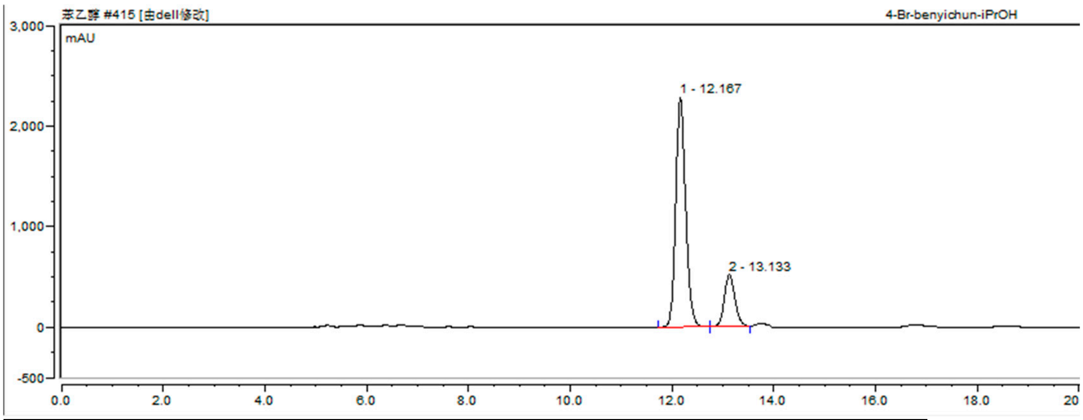

Peak#	Ret.time	Area	Height	Area%
1	12.167	513.9222	2283.057	80.72
2	13.133	122.7860	515.745	19.28

1-(naphthalen-2-yl)ethanol

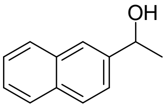

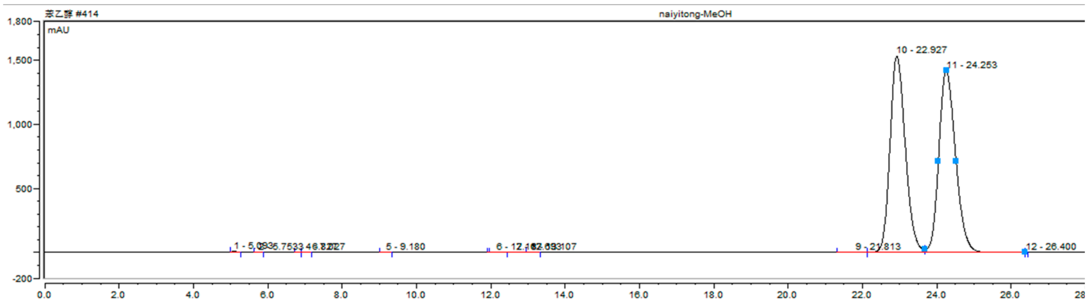

Peak#	Ret.time	Area	Height	Area%
1	22.927	729.8781	1531.927	49.79
2	24.253	736.6425	1419.507	50.21

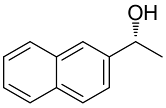

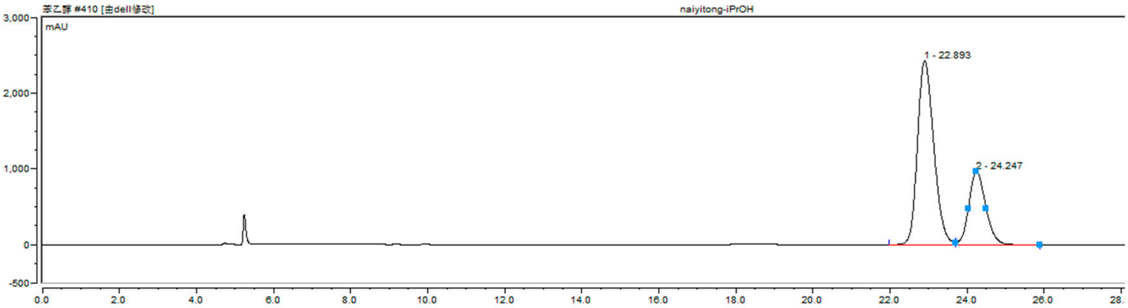

Peak#	Ret.time	Area	Height	Area%
1	22.893	1186.5369	2423.550	70.96
2	24.247	485.6739	960.067	29.04

1-([1,1'-biphenyl]-4-yl)ethanol

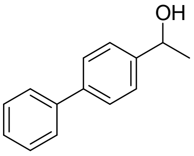

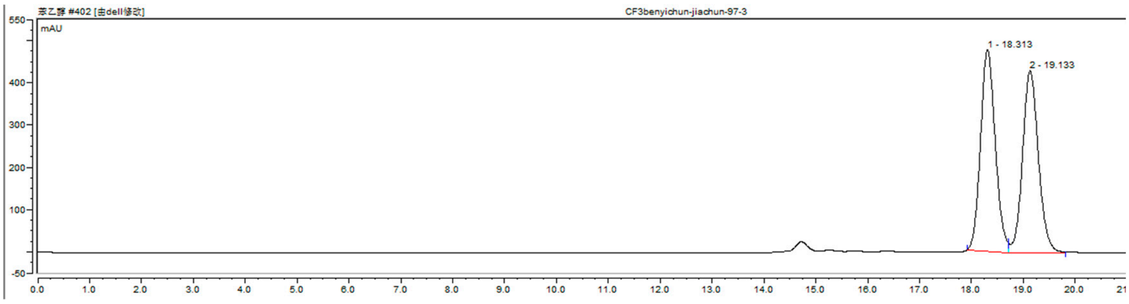

Peak#	Ret.time	Area	Height	Area%
1	18.313	157.0167	476.120	50.76
2	19.133	152.3106	430.212	49.24

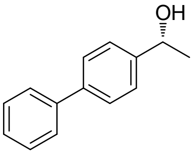

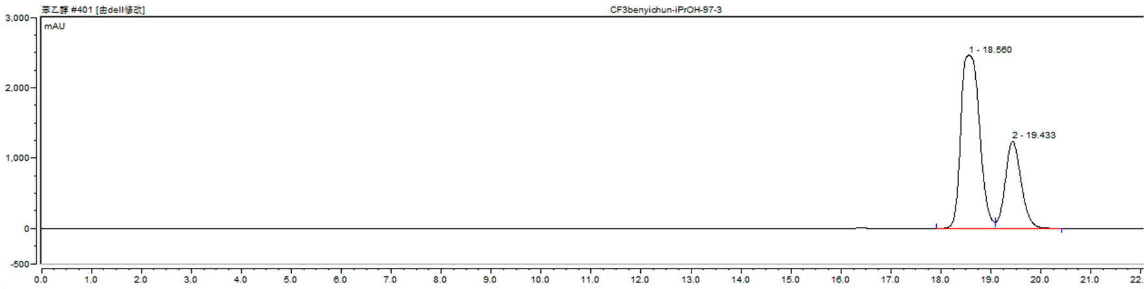

Peak#	Ret.time	Area	Height	Area%
1	18.560	1067.3390	2450.222	70.09
2	19.433	455.4359	1236.423	29.91

Reference

[1] Huang, M.; Cai, X.; Liu, Y.; Ke, Z. F. Base-controlled NHC-Ru-catalyzed transfer hydrogenation and α-methylation/transfer hydrogenation of ketones using methanol. *Chin. Chem. Lett.*, **2024**, *35*, 109323-109328.

[2] Aboo, A. H.; Begum, R.; Zhao, L.; Farooqi, Z. H.; Xiao, J. L. Methanol as hydrogen source: Chemoselective transfer hydrogenation of α,β-unsaturated ketones with a rhodacycle. *Chin. J. Catal.* **2019**, *40*, 1795-1799.

[3] Dey, S.; Thakur, S.D.; Sau, A.; Panja, D.; Roy, T.; Zhang, J.; Annadata, H.V.; Kundu, S. Cobalt catalyzed condensation interrupted selective transfer hydrogenation using methanol. *J. Catal.* **2024**, *439*, 115759–115769.

[4] Gaussian 16, Revision C.01, Frisch, M. J.; Trucks, G. W.; Schlegel, H. B.; Scuseria, G. E.; Robb, M. A.; Cheeseman, J. R.; Scalmani, G.; Barone, V.; Petersson, G. A.; Nakatsuji, H.; Li, X.; Caricato, M.; Marenich, A. V.; Bloino, J.; Janesko, B. G.; Gomperts, R.; Men Nucc, B.; Hratchian, H. P.; Ortiz, J. V.; Izmaylov, A. F.; Sonnenberg, J. L.; Williams-Young, D.; Ding, F.; Lipparini, F.; Egidi, F.; Goings, J.; Peng, B.; Petrone, A.; Henderson, T.; Ranasinghe, D.; Zakrzewski, V. G.; Gao, J.; Rega, N.; Zheng, G.; Liang, W.; Hada, M.; Ehara, M.; Toyota, K.; Fukuda, R.; Hasegawa, J.; Ishida, M.; Nakajima, T.; Honda, Y.; Kitao, O.; Nakai, H.; Vreven, T.; Throssell, K.; Montgomery, J. A., Jr.; Peralta, J. E.; Ogliaro, F.; Bearpark, M. J.; Heyd, J. J.; Brothers, E. N.; Kudin, K. N.; Staroverov, V. N.; Keith, T. A.; Kobayashi, R.; Normand, J.; Raghavachari, K.; Rendell, A. P.; Burant, J. C.; Iyengar, S. S.; Tomasi, J.; Cossi, M.; Millam, J. M.; Klene, M.; Adamo, C.; Cammi, R.; Ochterski, J. W.; Martin, R. L.; Morokuma, K.; Farkas, O.; Foresman, J. B.; Fox, D. J. Gaussian, Inc., Wallingford CT, **2016**.

[5] Zhao, Y.; Truhlar, D. G. A new local density functional for main-groupthermochemistry, transition metal bonding, thermochemicalkinetics, and noncovalent interactions. *J. Chem. Phys.* **2006**, *125*, 194101-194120.

[6] Raghavachari, K.; Binkley, J. S.; Seeger, R.; Pople, J. A. Self‐consistent molecular orbital methods. XX. A basis set for correlated wave functions. *J. Chem. Phys.* **1980**, *72*, 650-654.

[7] McLean, A. D.; Chandler, G. S. Contracted Gaussian basis sets for molecular calculations. I. Second row atoms, Z=11–18. *J. Chem. Phys.*, **1980**, *72*, 5639-5648.

[8] Clark, T.; Chandrasekhar, J.; Schleyer, P. v. R. Efficient diffuse function-augmented basis sets for anion calculations. III. The 3-21+G basis set for first-row elements, Li–F. *J. Comp. Chem.* **1983**, *4*, 294-302.

[9] Dolg, M.; Wedig, U.; Stoll, H.; Preuss, H. Energy-adjusted ab initio pseudopotentials for the first row transition elements. J. Chem. Phys. **1987**, *86*, 866-872.

[10] Bergner, A.; Dolg, M.; Kuechle, W.; Stoll, H.; Preuß, H. Ab initio energy-adjusted pseudopotentials for elements of groups 13–17. *Mol. Phys.* **1993**, *80*, 1431-1341.

[11] Fukui, K. Formulation of the reaction coordinate. *J. Phys. Chem.* **1970**, *74*, 4161.

[12] Fukui, K. The path of chemical reactions - the IRC approach. *Acc. Chem. Res.* **1981**, *14*, 363.

[13] Marenich, A. V.; Cramer, C. J.; Truhlar, D. G. Universal Solvation Model Based on Solute Electron Density and on a Continuum Model of the Solvent Defined by the Bulk Dielectric Constant and Atomic Surface Tensions. *J. Phys. Chem. B*. **2009**, *113*, 6378-6397.
